# FDX1 suppresses acute myeloid leukemia progression and reverses cuproptosis resistance via the ROS/p53/ME1 axis

**DOI:** 10.1016/j.isci.2026.117968

**Published:** 2026-10-26

**Authors:** Lihua Yao, Fangmin Zhong, Qin Bai, Huan Zeng, Yuxiang Qiu, Fangyi Yao, Yanmei Xu, Jing Zhang, Jin Lin, Keyu Deng, Bo Huang, Xiaozhong Wang

**Affiliations:** 1 Department of Clinical Laboratory, Jiangxi Province Key Laboratory of Immunology and Inflammation, Jiangxi Provincial Clinical Research Center for Laboratory Medicine, The Second Affiliated Hospital, Jiangxi Medical College, Nanchang University, Nanchang, Jiangxi 330006, P.R. China; 2 Department of Clinical Laboratory, Affiliated Hospital of North Sichuan Medical College, Department of Laboratory Medicine, North Sichuan Medical College, Nanchong, Sichuan 637000, P.R. China; 3 The National Engineering Research Center for Bioengineering Drugs and Technology, Institution of Translation Medicine, College of Life Science, College of Pharmacy, Nanchang University, Nanchang, Jiangxi 330031, P.R. China

**Keywords:** acute myeloid leukemia, AML, cuproptosis, FDX1, ME1, mitochondrial metabolism

## Abstract

Cuproptosis, a copper-dependent cell death mechanism, holds therapeutic potential in cancer, yet its regulatory pathways in acute myeloid leukemia (AML) remain poorly defined. We demonstrate that elevated serum copper levels correlate with poor prognosis in AML patients. Screening reveals that ferredoxin 1 (FDX1) is significantly downregulated in AML and associated with adverse outcomes. FDX1 loss promoted AML cell proliferation and cuproptosis resistance. Mechanistically, FDX1 enhanced mitochondrial damage and ROS/p53 signaling, which transcriptionally suppressed malic enzyme 1 (ME1). ME1 disrupted mitochondrial NAD^+^/NADH homeostasis and modulated lipoylated DLAT delipoylation, thereby limiting cuproptosis. ME1 was highly expressed in AML and supported leukemic cell growth, while its inhibition restored cuproptosis sensitivity in FDX1-low cells. Given the elevated serum copper levels in AML and the capacity of ME1 inhibition to reverse cuproptosis resistance induced by low FDX1 expression, the combination of copper ionophores with ME1 inhibitors may represent a potential therapeutic strategy for AML.

## Highlights

• Elevated serum copper and low FDX1 expression are linked to poor AML outcomes
• FDX1 sensitizes AML cells to cuproptosis through ROS/p53 signaling
• p53 directly represses ME1 to connect mitochondrial stress with cuproptosis
• ME1 inhibition restores cuproptosis sensitivity and enhances elesclomol efficacy

## Graphical abstract

**Figure:**
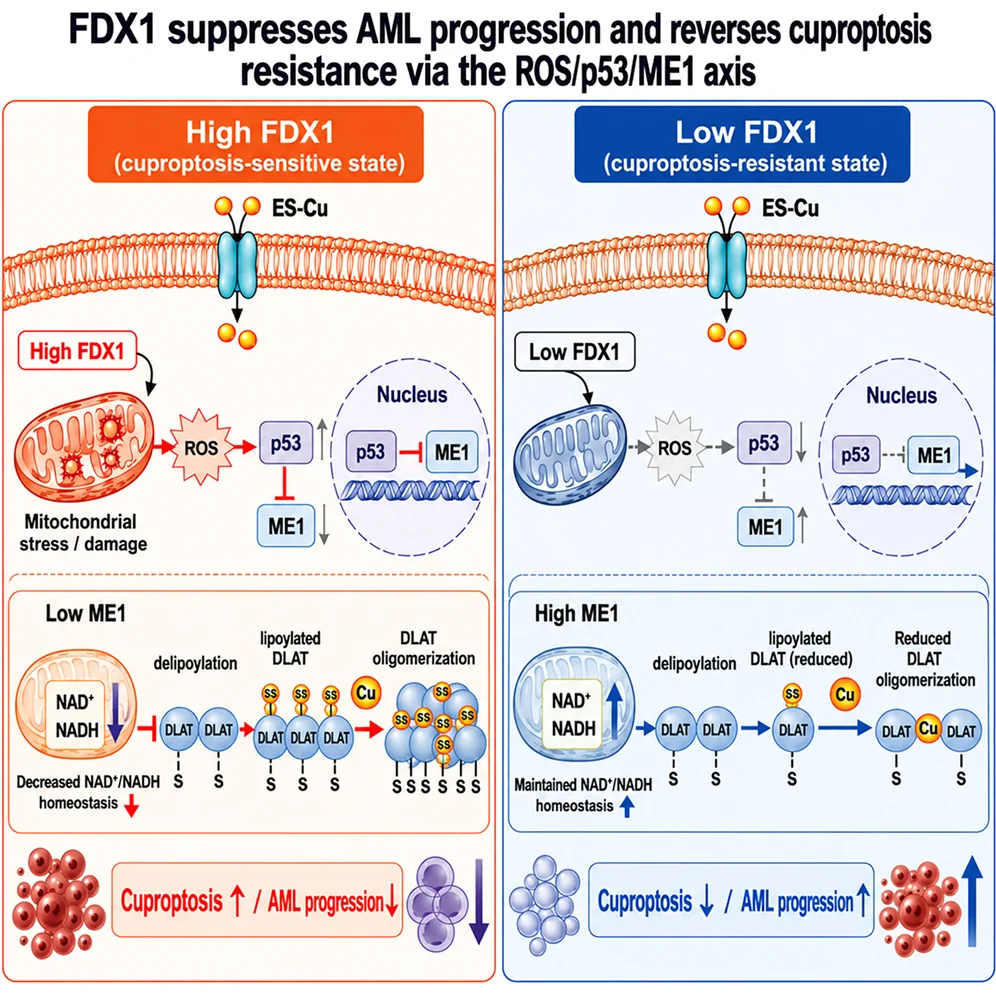

## Teaser

molecular biology; molecular network; cancer

## Introduction

Acute myeloid leukemia (AML) is a highly aggressive hematologic malignancy characterized by rapid progression and poor clinical outcomes. Current treatment strategies for AML primarily rely on intensive chemotherapy regimens, including cytosine arabinoside and anthracyclines, often combined with hypomethylating agents.^1^ Although novel targeted therapies, such as ivosidenib (IDH1 inhibitor), enasidenib (IDH2 inhibitor), gilteritinib (FLT3 inhibitor), and venetoclax (BCL-2 inhibitor), have shown promise,^2^^,^^3^^,^^4^^,^^5^ the five-year overall survival (OS) rate remains dismal, with the 5-year OS rate for relapsed/refractory AML being approximately 10%.^6^ The majority of patients ultimately succumb to disease recurrence or progression, driven by treatment resistance or severe side effects. Thus, there is an urgent need to develop novel and effective therapeutic strategies to improve outcomes in AML.

Copper, an essential trace element, serves as a critical cofactor for numerous enzymes and plays a pivotal role in various biological redox processes.^7^ However, excessive copper accumulation can exert significant toxicity. Emerging evidence has highlighted a complex relationship between copper dysregulation and AML progression.^8^^,^^9^^,^^10^^,^^11^ Notably, elevated serum copper levels are strongly associated with inferior OS and reduced disease-free survival, suggesting its prognostic significance in AML.^11^ Recent studies have identified that aberrant copper accumulation induces a unique form of cell death termed cuproptosis, which is distinct from other well-characterized cell death mechanisms, such as apoptosis or ferroptosis.^12^ Growing evidence suggests that cuproptosis contributes to tumor progression and therapeutic response in multiple cancer types.^13^^,^^14^^,^^15^^,^^16^^,^^17^^,^^18^^,^^19^ Importantly, emerging evidence has begun to link cuproptosis directly to AML. Lewis et al. recently reported that inhibition of heme biosynthesis triggers cuproptosis in AML, suggesting that mitochondrial copper-dependent proteotoxic stress may represent a therapeutically exploitable vulnerability in leukemia cells.^20^ Given that cuproptosis is mediated by mitochondrial proteotoxic stress, the high dependence of AML cells on mitochondrial respiration may create a therapeutic vulnerability to cuproptosis induction.^21^ Nevertheless, the mechanisms determining cuproptosis sensitivity or resistance in AML remain incompletely understood.

AML cells are characterized by altered mitochondrial metabolism and redox homeostasis, and ROS have been implicated in leukemic transformation, AML cell survival, metabolic remodeling, and therapeutic response.^22^^,^^23^ Excessive ROS accumulation can induce mitochondrial damage and activate stress-responsive pathways, among which p53 is a central regulator of cell death, redox homeostasis, and metabolic reprogramming.^24^ Recent evidence further suggests that p53 may participate in the regulation of cuproptosis-related metabolic cell death.^25^ FDX1, a mitochondrial ferredoxin, has been identified as a key regulator of protein lipoylation and copper ionophore-induced cuproptosis.^12^^,^^26^ However, whether FDX1 dysregulation contributes to cuproptosis resistance in AML, and whether ROS/p53 signaling is involved in this process, remains unclear.

ME1 has recently emerged as an important metabolic regulator in AML. High ME1 expression has been reported to predict adverse prognosis in cytogenetically normal AML and to promote leukemogenesis through the IL-6/JAK2/STAT3 pathway.^27^ In addition, NRF2 has been shown to maintain redox balance by transcriptionally upregulating ME1 in NPM1-mutated AML, and NRF2 inhibition synergizes with venetoclax.^28^ These findings suggest that ME1 may serve as an important metabolic and redox-regulatory node in AML. However, whether ME1 participates in the regulation of cuproptosis sensitivity, and how it may be linked to FDX1-mediated mitochondrial stress, remain unknown.

In this study, we observed a significant elevation in serum copper levels in AML patients, with higher levels correlating with worse OS. Among cuproptosis-related genes (CRGs), FDX1 was the most significantly downregulated in AML compared to healthy controls. Functional studies revealed that FDX1 downregulation promotes AML cell proliferation and cuproptosis resistance. Conversely, FDX1 overexpression inhibits cell proliferation and markedly enhances AML cell sensitivity to cuproptosis. Mechanistically, FDX1 exacerbates mitochondrial damage and promotes ROS accumulation, thereby activating p53, which transcriptionally represses ME1. ME1 inhibition disrupts mitochondrial NAD^+^/NADH homeostasis and impairs lipoylated DLAT delipoylation, thereby enhancing sensitivity to cuproptosis. ME1 is highly expressed in AML patients and promotes AML cell proliferation. Thus, leveraging endogenous high copper levels and targeting ME1 to induce cuproptosis may offer a promising new approach for AML treatment.

## Results

### Screening of the cuproptosis-related gene FDX1 is associated with the prognosis of AML patients

Elevated intracellular copper levels trigger cuproptosis, yet the precise regulatory pathways involved in tumor cuproptosis are still not fully understood.^12^ We first measured serum copper levels in AML patients and found that serum copper concentrations were higher than normal levels (Figure 1A). Moreover, elevated serum copper levels were inversely associated with OS in AML patients, suggesting that increased serum copper may be associated with poor prognosis in AML (Figure 1B). We further analyzed whether serum copper level was associated with AML subtype. As shown in Table S2, the FAB subtype distribution did not differ significantly between patients with normal and high serum copper levels in our clinical cohort. To assess the impact of copper ions on AML cells, we exposed them to copper and copper ionophore (elesclomol) (Figures 1C–1G), indicating that AML cells are sensitive to copper and their responsiveness is dose-dependent on the copper. Notably, when treated with a cuproptosis inhibitor (tetrathiomolybdate, TTM), the effect of ES-Cu on AML cells was reversed.

**Figure 1:**
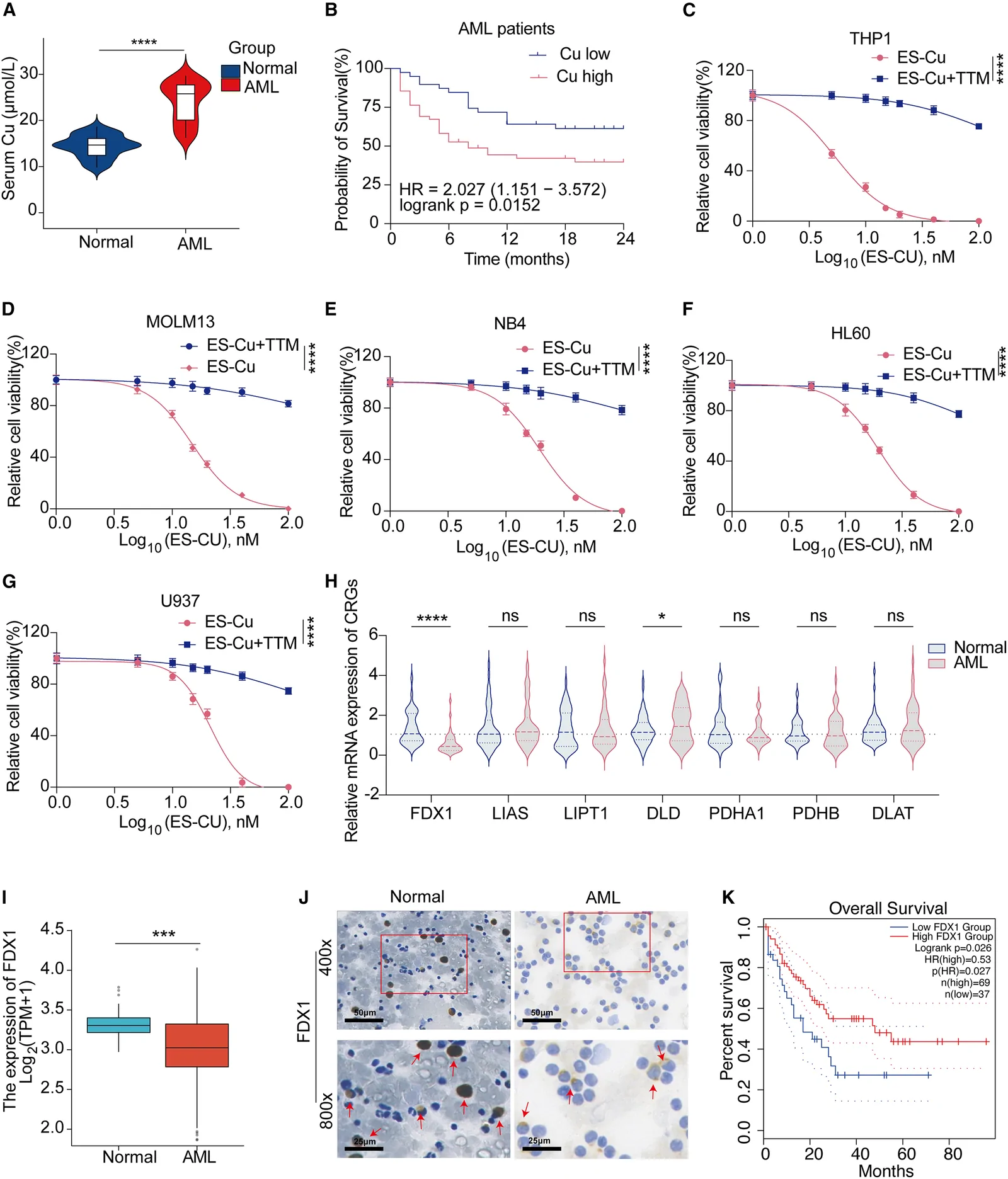
Screening of the cuproptosis-related gene FDX1 is associated with the prognosis of AML patients (A) Difference in serum Cu concentrations between patients with AML and healthy controls (AML *n* = 126, normal *n* = 76 biologically independent samples). (B) Among the AML patients with available survival information, 55 and 39 patients were classified into the high- and low-serum-Cu groups, respectively. OS was compared between patients with high and low serum Cu concentrations. (C–G) ES-Cu reduced AML cell viability in a dose-dependent manner, and TTM reversed this effect. (H) The mRNA levels of cuproptosis-related genes in PBMCs from primary AML patients compared with PBMCs from healthy controls (AML *n* = 55, normal *n* = 40 biologically independent samples). (I) The mRNA expression of FDX1 in normal group and AML patients from XENA database. (J) Representative IHC images show FDX1 protein expression in bone marrow (BM) from primary AML patients (*n* = 11) and normal BM (*n* = 10). Scale bars: 50 μm (upper), 25 μm (lower). (K) OS analysis of the effect of FDX1 expression patterns in the AML patients from GEPIA database. Student’s *t* test was used in (A and I), survival curves were estimated using the Kaplan-Meier method and compared using a two-sided log rank test in (B and K), two-way analysis of variance (ANOVA) was used in (C–G), two-tailed Mann-Whitney U test was used in (H). ns. represents not significant. ∗*p* < 0.05, ∗∗*p* < 0.01, and ∗∗∗*p* < 0.001 vs. control.

To date, seven genes (FDX1, LIPT1, LIAS, PDHB, DLD, PDHA1, and DLAT) related to cuproptosis have been reported.^12^ We detected the mRNA expression profiles of CRGs by RT-PCR in peripheral blood mononuclear cells (PBMCs) from AML patients and compared them with those from healthy controls. Among these genes, FDX1 exhibited the most significant differential expression in AML patients (Figures 1H and 1I). However, FDX1 expression status was not significantly associated with FAB subtype distribution in our clinical cohort (Table S3). Moreover, IHC staining revealed reduced FDX1 protein expression in AML bone marrow samples compared with normal controls (Figure 1J). Furthermore, lower FDX1 expression correlated with a worse prognosis in AML patients (Figure 1K). These findings suggested that low expression of FDX1 positively correlated with an unfavorable prognosis in AML patients.

### FDX1 inhibited the growth of AML cells

To investigate the functional role of FDX1 in AML cells, we analyzed FDX1 expression across AML cell lines using the CCLE/DepMap gene expression dataset (Figure S2). The results showed heterogeneous FDX1 expression among AML cell lines. MOLM13 and THP1 exhibited detectable but relatively low-to-moderate FDX1 expression, consistent with the reduced FDX1 expression observed in primary AML samples. We established FDX1-knockdown (FDX1-KD) and FDX1-overexpressing (FDX1-OE) models in THP1 and MOLM13 cell lines (Figures 2A and 2B). CCK-8 assays demonstrated that FDX1 overexpression significantly suppressed AML cell viability (Figure 2C), whereas FDX1 depletion markedly enhanced the proliferation of THP1 and MOLM13 cells (Figure 2D). Next, Cell cycle analysis revealed that FDX1-upregulated AML cells exhibited a decreased proportion of S-phase cells and a concurrent increase in G0/G1-phase cells (Figure 2E). Conversely, FDX1 knockdown induced opposite effects characterized by reduced G0/G1 phase and elevated S phase distribution (Figure 2F).

**Figure 2:**
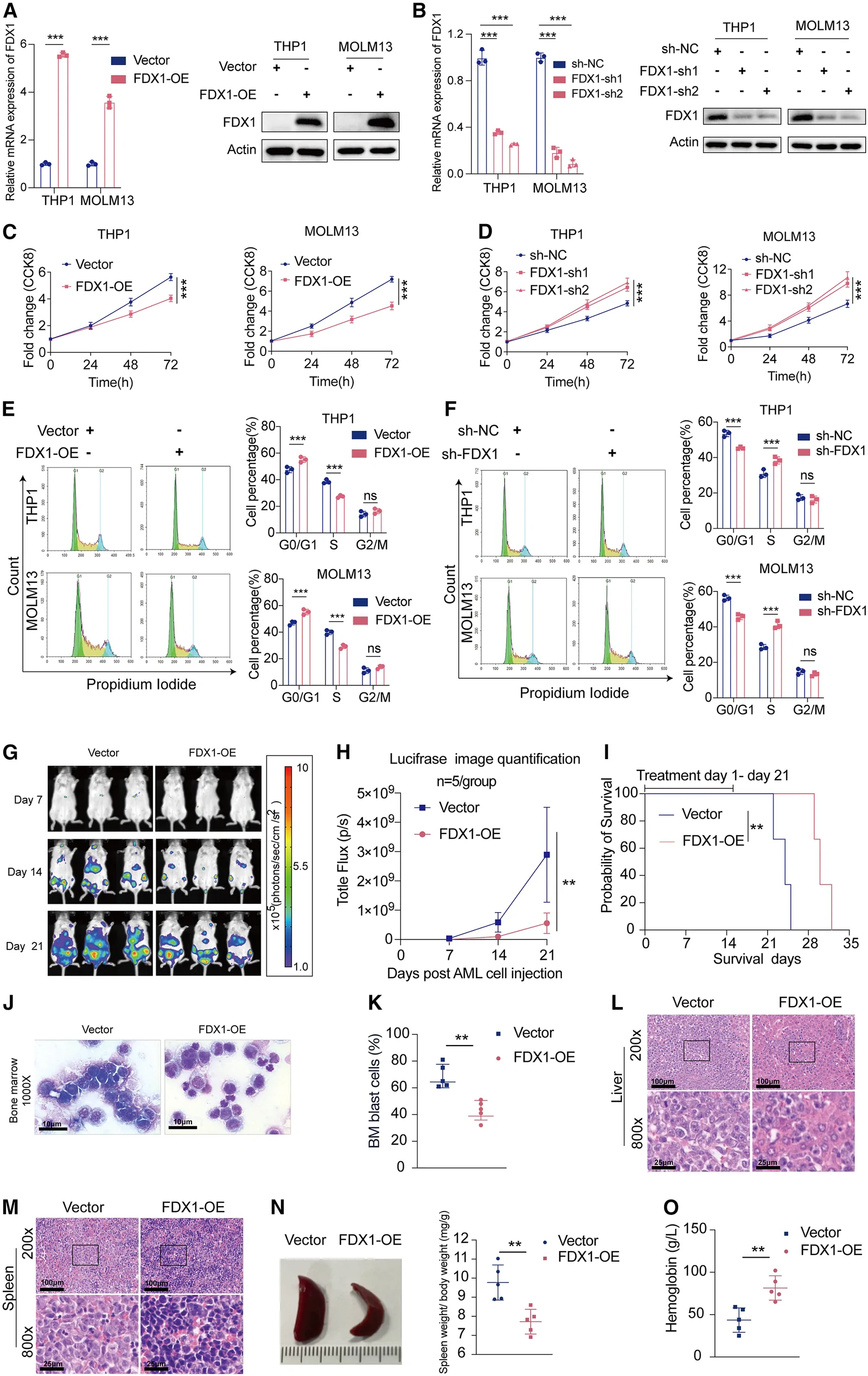
FDX1 inhibited the growth of AML cells (A and B) RT-qPCR and western blot analysis of both FDX1 mRNA levels and FDX1 protein levels in FDX1-overexpression and FDX1 knockdown AML cells. (C and D) Cellular viability was quantitatively assessed using CCK-8 assays at specified time points (0 h, 24 h, 48 h, 72 h) following stable overexpression of FDX1 (FDX1-OE) and FDX1 knockdown (FDX1-KD) in THP1 and MOLM13 cells. (E and F) The cell cycle measured by flow cytometry in stable FDX1-overexpression THP1 and MOLM13 cells. (G and H) Tumor growth in NSG mice harboring FDX1-OE MOLM13 cells was monitored weekly by bioluminescence imaging (G) and the quantification of luciferase signals for all mice per group (H). (I) Kaplan-Meier analysis of survival of NSG mice. (J and K) Percentage of blasts in the bone marrow of leukemic NSG mice after 3 weeks. Cells from bone marrow of mice were tested by Wright-Giemsa staining. Scale bars: 10 μm. (L and M) HE staining of mouse liver and spleen tissues in leukemic NSG mice. Scale bars: 100 μm (upper), 25 μm (lower). (N and O) Spleen weight (N) and hemoglobin levels (O) in leukemic NSG mice after 3 weeks. Data are presented as mean ± SD from three independent experiments or five mice per group. Student’s *t* test was used in (A, E, F, K, N, and O), one-way ANOVA was used (B), two-way ANOVA was used in (C, D, and H), and survival curves were estimated using the Kaplan-Meier method and compared using a two-sided log rank test in (I). ∗∗*p* < 0.01, and ∗∗∗*p* < 0.001 vs. control; ns, not significant.

Based on *in vitro* studies demonstrating that upregulation of FDX1 inhibits AML cell proliferation, we successfully established an AML mouse model to further investigate the role of FDX1 *in vivo*. Luciferase-expressing MOLM13 cells stably overexpressing FDX1 were injected into NSG mice via the tail vein, allowing longitudinal monitoring of leukemic engraftment and disease burden by bioluminescence imaging (Figures 2G and 2H). In accordance with our *in vitro* observations, mice harboring AML cells with overexpressed FDX1 exhibited reduced leukemia burden and longer OS (Figure 2I). Furthermore, compared with the control group, AML cell infiltration was reduced in the bone marrow (Figures 2J and 2K), liver (Figure 2L), and spleen (Figure 2M) of mice, accompanied by alleviation of splenomegaly (Figure 2N) and anemia (Figure 2O) in the overexpression group. Overall, FDX1 overexpression inhibited AML cell growth and significantly reduced leukemia burden *in vivo*.

### FDX1 overexpression enhances AML cell sensitivity to cuproptosis

The role of FDX1 expression, a key gene in the cuproptosis pathway, in modulating cuproptosis within AML is currently unknown. Because ES-Cu inhibited AML cell viability, we next investigated whether FDX1 expression affected cellular sensitivity to ES-Cu. FDX1 overexpression enhanced ES-Cu-induced cell death (Figure 3A), whereas FDX1 knockdown reduced it compared with the corresponding control (Figure 3B). Furthermore, the elevated expression level of FDX1 increased ES-Cu-induced cell death (Figure 3C), while cell death induced by ES-Cu was reduced in the FDX1-knockdown AML cells, compared with the control group (Figure 3D). To further distinguish ES-Cu-induced cell death from apoptosis and ferroptosis, we performed inhibitor-based control experiments using different cell death inducers and inhibitors. ES-Cu-induced cell death was rescued by the copper chelator TTM, but not by the ferroptosis inhibitor Fer-1 or the apoptosis inhibitors Z-VAD-FMK and BOC-D-FMK. These results indicate that ES-Cu predominantly induces copper-dependent cuproptosis in AML cells rather than apoptosis- or ferroptosis-dominant cell death (Figures S3A and S3B). To further confirm the activation of cuproptosis, we assessed additional cuproptosis-related markers. In addition to increased DLAT oligomerization, FDX1-overexpressing AML cells treated with ES-Cu showed increased lipoylated-DLAT levels, whereas FDX1 knockdown attenuated DLAT oligomerization and lipoylated-DLAT levels (Figure 3E). Furthermore, treatment with the copper chelator TTM partially rescued ES-Cu-induced growth inhibition in FDX1-overexpressing AML cells (Figures S3C and S3D).

**Figure 3:**
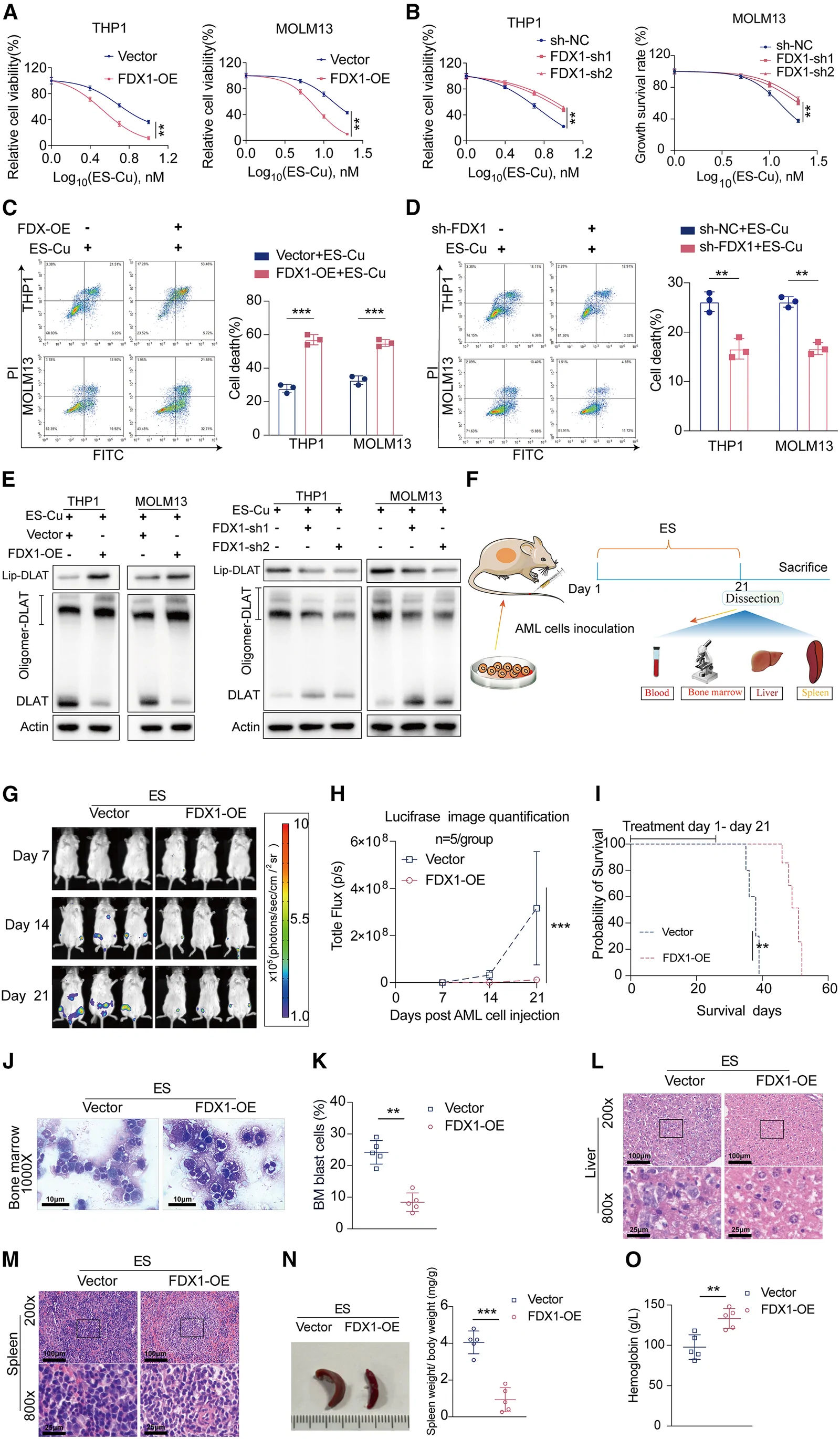
FDX1 overexpression enhances AML cell sensitivity to cuproptosis (A and B) FDX1-OE and FDX1-KD AML cells were treated with indicated concentrations of ES-Cu (1:1 ratio) for 48 h, cell viability was subsequently detected by the CCK-8 assay. (C and D) Cell death rate measured by flow cytometry in stable FDX1-overexpression THP1 and MOLM13 cells with ES-Cu treatment for 48 h. (E) Lipoylated DLAT(Lip-DLAT) and DLAT oligomerization were analyzed by western blot in stable FDX1-overexpression and FDX-knockdown THP1 (ES-Cu, 5 nM) and MOLM13 (ES-Cu, 10 nM) cells after 48 h. (F) The scheme outlines the tail vein injection of either vector-control MOLM13 cells or FDX1-OE MOLM13 cells into NSG mice, which is subsequently followed by treatment with ES (10 mg/kg). (G and H) Tumor growth in NSG mice harboring FDX1-OE MOLM13 cells was monitored weekly by bioluminescence imaging (G) and the quantification of luciferase signals for all mice per group (H). (I) Kaplan-Meier analysis of survival of NSG mice. (J and K) Percentage of blasts in bone marrow in leukemic NSG mice after 3 weeks. Cells from bone marrow of mice were tested by Wright-Giemsa staining. Scale bars: 10 μm. (L and M) HE staining of mouse liver and spleen tissues in leukemic NSG mice after 3 weeks treatment. Scale bars: 100 μm (upper), 25 μm (lower). (N and O) Spleen weight (N) and hemoglobin levels (O) in leukemic NSG mice after 3 weeks treatment. Data are presented as mean ± SD from three independent experiments or five mice per group. Two-way ANOVA was used in (A–D) and (H), student’s *t* test was used in (K, N, and O), and survival curves were estimated using the Kaplan-Meier method and compared using a two-sided log rank test in (I). ∗∗*p* < 0.01, and ∗∗∗*p* < 0.001 vs. control.

To substantiate these observations *in vivo*, we employed an AML mouse model treated with the copper ionophore elesclomol (Figure 3F). The results demonstrated that in mice bearing FDX1-overexpressing AML cells, elesclomol treatment significantly reduced leukemia burden (Figures 3G and 3H) and prolonged OS (Figure 3I), compared with the control group. Furthermore, treatment with elesclomol effectively suppressed the infiltration of FDX1-overexpressing AML cells into the bone marrow (Figures 3J and 3K), liver and spleen (Figures 3L and 3M) of mice, while alleviating splenomegaly (Figure 3N) and restoring peripheral blood hemoglobin levels to near-physiological levels (Figure 3O). Collectively, these findings demonstrate that FDX1 enhances AML cell sensitivity to cuproptosis and suppresses AML progression.

### FDX1 overexpression disrupts mitochondrial function

Accumulating evidence indicates that the stabilization of mitochondrial function is pivotal for the tumor stemness and the progression of tumorigenesis.^29^^,^^30^ FDX1, a mitochondrial iron-sulfur protein, participates in electron transport. Furthermore, as a key upstream regulator of cuproptosis, FDX1 enhances the toxic potential of copper ions by catalyzing the reduction of Cu^2+^ to Cu^+^.^31^ To define the effects of FDX1 on mitochondrial structure and function, we examined mitochondrial ultrastructure, membrane potential, and intracellular ROS levels in THP1 and MOLM13 cells. TEM showed that FDX1 overexpression alone caused partial cristae fragmentation and a reduction in cristae number. Following ES-Cu treatment, mitochondrial swelling, cristae loss, and vacuolization were more pronounced in FDX1-overexpressing cells than in vector-control cells (Figure 4A). Quantitative analysis confirmed that FDX1 overexpression increased the percentage of mitochondria with abnormal ultrastructure and further exacerbated ES-Cu-induced mitochondrial damage (Figure 4B). Consistent with these ultrastructural alterations, JC-1 staining revealed decreased red aggregate fluorescence and increased green monomer fluorescence in FDX1-overexpressing cells, indicating a loss of MMP. These changes were more pronounced following ES-Cu treatment (Figures 4C and 4D). Flow cytometric analysis and quantification of JC-1 aggregates further confirmed that FDX1 overexpression reduced MMP in both THP1 and MOLM13 cells and enhanced ES-Cu-induced mitochondrial depolarization (Figures 4E and 4F). Given that mitochondrial damage can trigger ROS and the activation of cell death pathways,^32^ we measured cellular ROS levels and found that FDX1 overexpression promoted ROS production in AML cells. Under equivalent ES-Cu exposure, FDX1 overexpression exacerbated ROS accumulation (Figures 4G and 4H). Collectively, these findings indicate that FDX1 overexpression disrupts mitochondrial homeostasis, reduces MMP, and increases ROS accumulation, thereby sensitizing AML cells to ES-Cu-induced mitochondrial injury.

**Figure 4:**
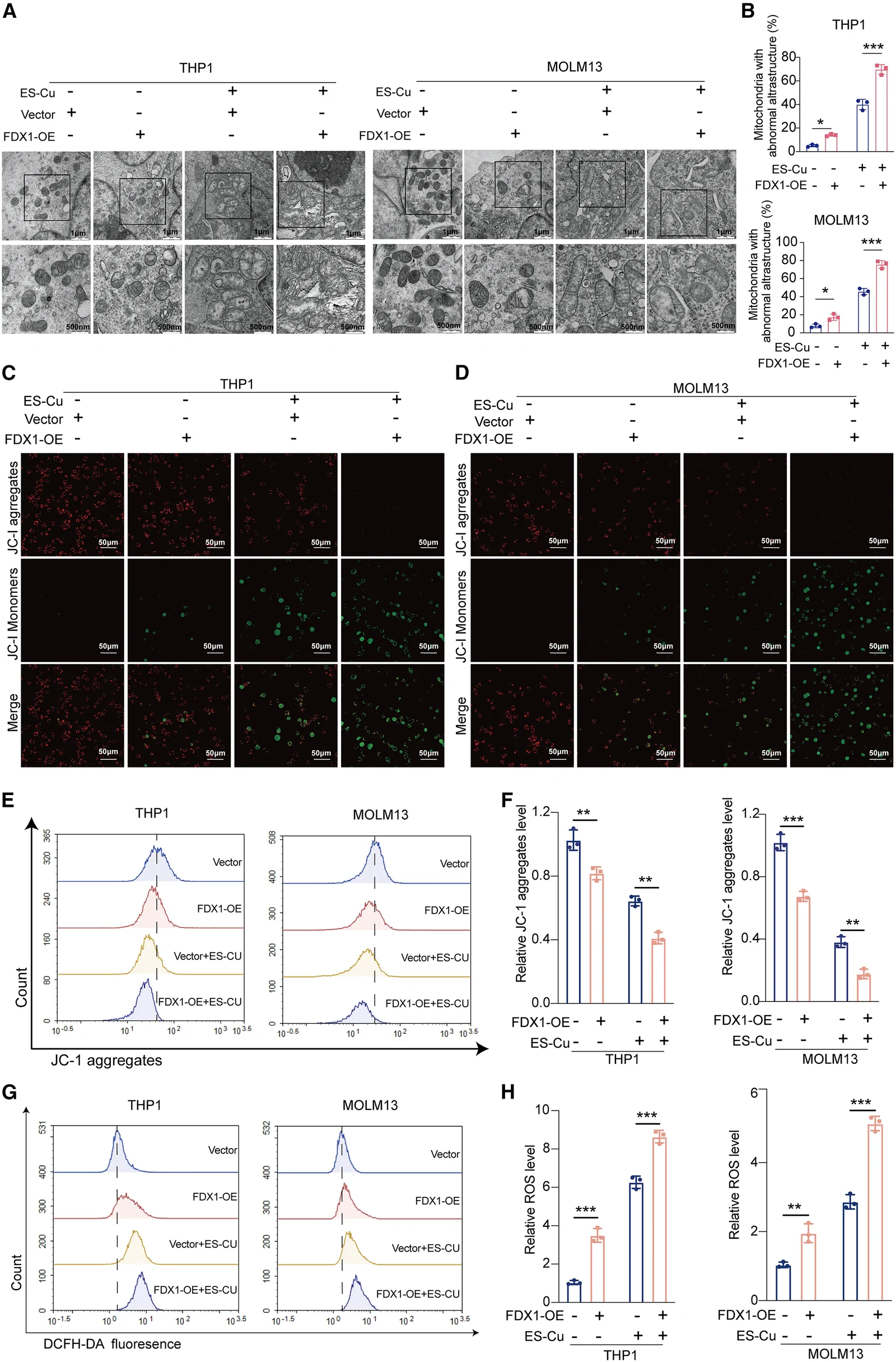
FDX1 overexpression disrupts mitochondrial function (A) Representative TEM images of stable FDX1-overexpressing THP1 and MOLM13 cells treated with or without ES-Cu for 48 h. Scale bars: 1 μm (upper), 500 nm (lower). (B) Quantification of the percentage of mitochondria with abnormal ultrastructure in experiments as in (A) (*n* = 100 mitochondria per condition). (C and D) Confocal microscopy analysis of changes in JC-1 green monomer and red aggregate fluorescence in FDX1-overexpressing THP1 and MOLM13 cells following 48-h treatment with or without ES-Cu. Scale bars, 50 μm. (E and F) Mitochondrial membrane potential was assessed by JC-1 staining and quantified using flow cytometry. (G and H) The ROS levels were measured by flow cytometry. Data are presented as mean ± SD from three independent experiments. Two-way ANOVA was used in (B, F, and H). ∗*p* < 0.05, ∗∗*p* < 0.01, and ∗∗∗*p* < 0.001 vs. control.

### FDX1 induces activation of the p53/ME1 axis via ROS

To elucidate the mechanisms underlying cuproptosis resistance induced by FDX1 depletion in AML, we conducted comprehensive RNA sequencing analysis and non-targeted metabolomic profiling on MOLM13 cells with FDX1 knockdown. The subsequent KEGG analysis of the DEGs revealed that the absence of FDX1 significantly altered the expression patterns of genes associated with DNA damage and the p53 signaling pathway (Figure 5A). As a key transcription factor, p53 regulates diverse target genes governing cell cycle progression, senescence, cell death, and cell metabolism. Intriguingly, KEGG enrichment analysis of cellular processes based on differential metabolites unveiled a significant association between FDX1 deficiency and pyruvate metabolism (Figures 5B and 5C). FDX1 knockdown substantially elevated pyruvate levels in AML cells compared to controls (Figure 5D), whereas FDX1 overexpression reduced pyruvate accumulation (Figure 5E), prompting our hypothesis that p53 activation modulates pyruvate metabolic genes in AML cells. To investigate p53-mediated regulation of pyruvate metabolism, we cross-referenced p53 ChIP-seq data (GSM2501568), RNA-seq DEGs, and pyruvate metabolism-related genes, identifying ME1 as a potential downstream target of p53 (Figure 5F). Consistent with this, ME1 expression was markedly upregulated in FDX1 knockdown cells (Figures 5G and 5H). Knockdown of p53 in both MOLM13 and THP1 cells led to an increase in both mRNA and protein levels of ME1 (Figures 5I and 5J), thereby providing further support for our hypothesis. The JASPAR database revealed putative p53 binding motifs within the ME1 promoter region (Figure 5K), which were experimentally confirmed via chromatin immunoprecipitation (ChIP), and ChIP-PCR assays demonstrated direct p53 binding to the ME1 promoter (Figures 5L and 5M). Together with previous evidence showing that p53 represses ME1 expression,^33^ these findings support that p53 transcriptionally regulates ME1 expression through direct promoter binding.

**Figure 5:**
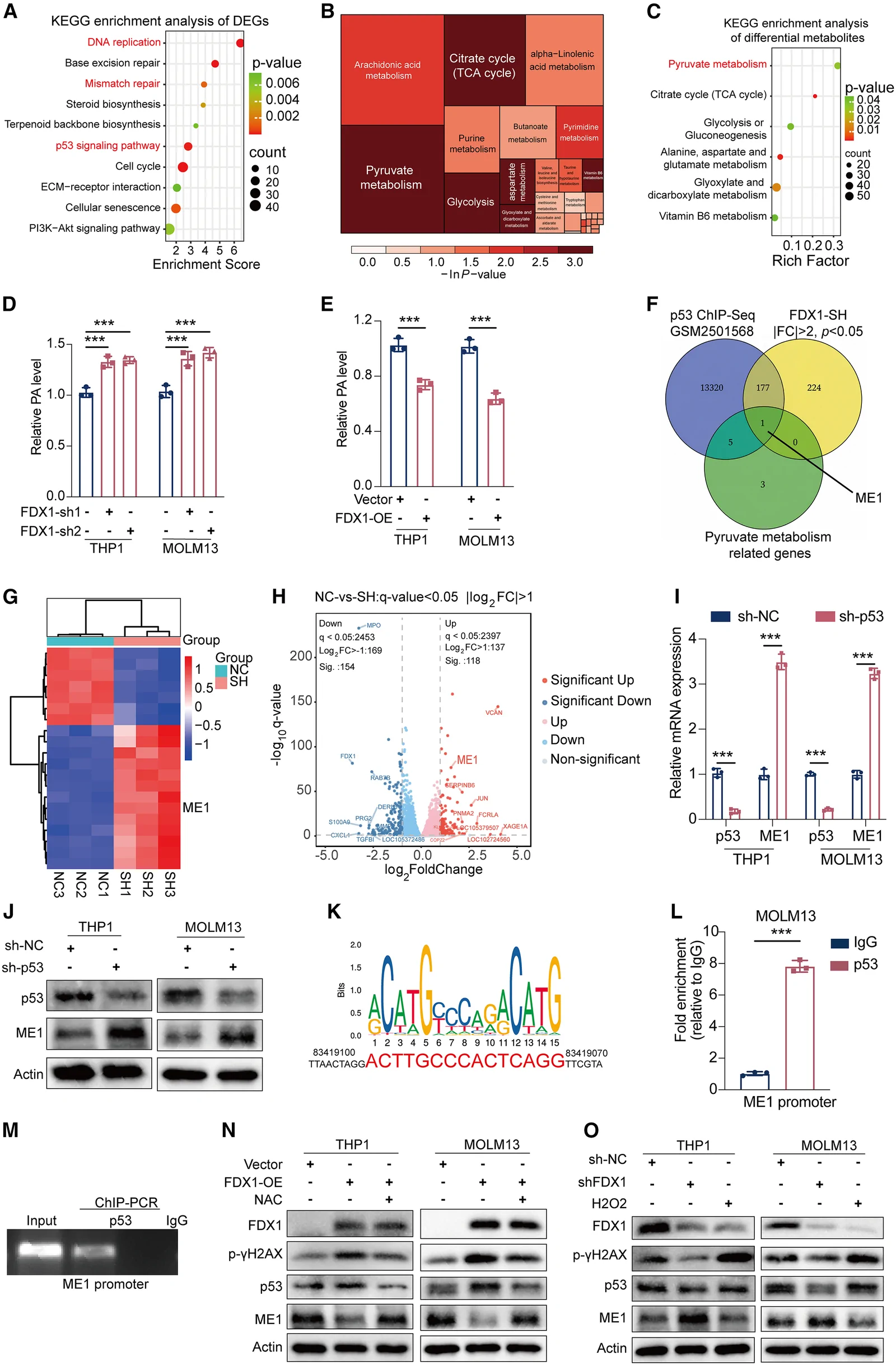
FDX1 induces activation of the p53/ME1 axis via ROS (A) KEGG analysis was conducted on RNA-seq DEGs (*p* < 0.05, fold change ≥1.5) identified between FDX1 knockdown MOLM13 cells and control cells. (B) Hierarchical clustering heatmap depicting differential metabolites in MOLM13 cells following FDX1 knockdown. (C) KEGG analysis of metabolic pathways was conducted based on differential metabolites identified by non-targeted metabolomics. (D and E) Pyruvic acid levels detected by pyruvate (PA) content assay kit. (F) Venn diagram depicting the genes at the intersection of p53 ChIP-seq data, RNA-seq data from FDX1 knockdown MOLM13 cells, and a set of pyruvate-regulated genes. (G) The heatmap depicting the expression levels of genes in control and FDX1 knockdown MOLM13 cells. (H) The volcano map depicting the expression levels of genes in control and FDX1 knockdown MOLM13 cells. (I and J) The mRNA and protein levels of ME1 and p53 in knockdown p53 AML cells were detected by RT-qPCR and western blot, respectively. (K) p53 DNA-binding sites are presented in the human ME1 promoter region. (L and M) ChIP-PCR in MOLM13 cells demonstrated ME1 promoter occupancy by p53. (N and O) In FDX1 overexpressing or knockdown cells, the levels of p53,ME1 and p-γH2AX were determined by western blot after treatment with NAC (5 mM) or H_2_O_2_ (10 μM). Data are presented as mean ± SD from three independent experiments. One-way ANOVA was used (B), Student’s *t* test was used in (E, I, and L). ∗∗*p* < 0.01 and ∗∗∗*p* < 0.001 vs. control.

Mitochondrial metabolic dysfunction generates substantial reactive oxygen species (ROS), encompassing superoxide anion (O_2_ · −), hydrogen peroxide (H_2_O_2_), hydroxyl radicals (·OH), and singlet oxygen (^1^O_2_).^34^ Excessive ROS accumulation induces mitochondrial damage, depolarization, and DNA lesions,^35^ while concurrently activating the p53 signaling pathway.^24^ We therefore propose that FDX1 elevates ROS levels to activate p53, which transcriptionally represses its target gene ME1. The results revealed elevated p53 expression, reduced ME1 levels, and increased DNA damage marker p-γH2AX in FDX1-overexpressing cells. To validate this mechanism, treatment with the antioxidant N-acetylcysteine (NAC) reversed this effect by abolishing the elevated ROS levels (Figure 5N). Conversely, FDX1 knockdown decreased ROS production, downregulated p53, increased ME1 expression, and attenuated p-γH2AX expression. Strikingly, supplementation with H_2_O_2_ restored p53/ME1 axis activation and exacerbated DNA damage in FDX1-deficient cells (Figure 5O). These findings collectively demonstrate that FDX1 governs p53 activation via ROS generation, enabling p53-mediated transcriptional regulation of its downstream target ME1.

### ME1 promotes resistance to cuproptosis by disrupting mitochondrial NAD^+^/NADH homeostasis

ME1 is a pivotal cytoplasmic metabolic enzyme that catalyzes the decarboxylation of malate to pyruvate.^36^ Recent studies have revealed that ME1 is highly overexpressed in various cancers and is closely associated with tumor proliferation, metastasis, and therapy resistance.^37^^,^^38^^,^^39^ However, the functional significance of ME1 in AML remains underexplored. ME1 mRNA and protein levels were higher in AML patients compared to the normal group (Figures 6A and 6B). Moreover, higher ME1 expression levels were associated with a poorer OS rate in AML patients (Figure 6C). We constructed and validated ME1-knockdown and ME1-overexpressing AML cell lines (Figures 6D and 6E). Knockdown of ME1 resulted in a pronounced reduction of cell viability in AML cells (Figure 6F). Treatment with the ME1 inhibitor similarly reduced AML cell viability (Figure 6G). Conversely, ME1 overexpression enhanced AML cell viability (Figure 6H). We subsequently analyzed the effects of ME1 inhibition on ES-Cu sensitivity in AML cells. Notably, suppression of ME1 significantly enhanced ES-Cu induced cuproptosis (Figures 6I and 6J), while ME1 upregulation conferred increased resistance to cuproptosis in AML cells (Figure 6K).

**Figure 6:**
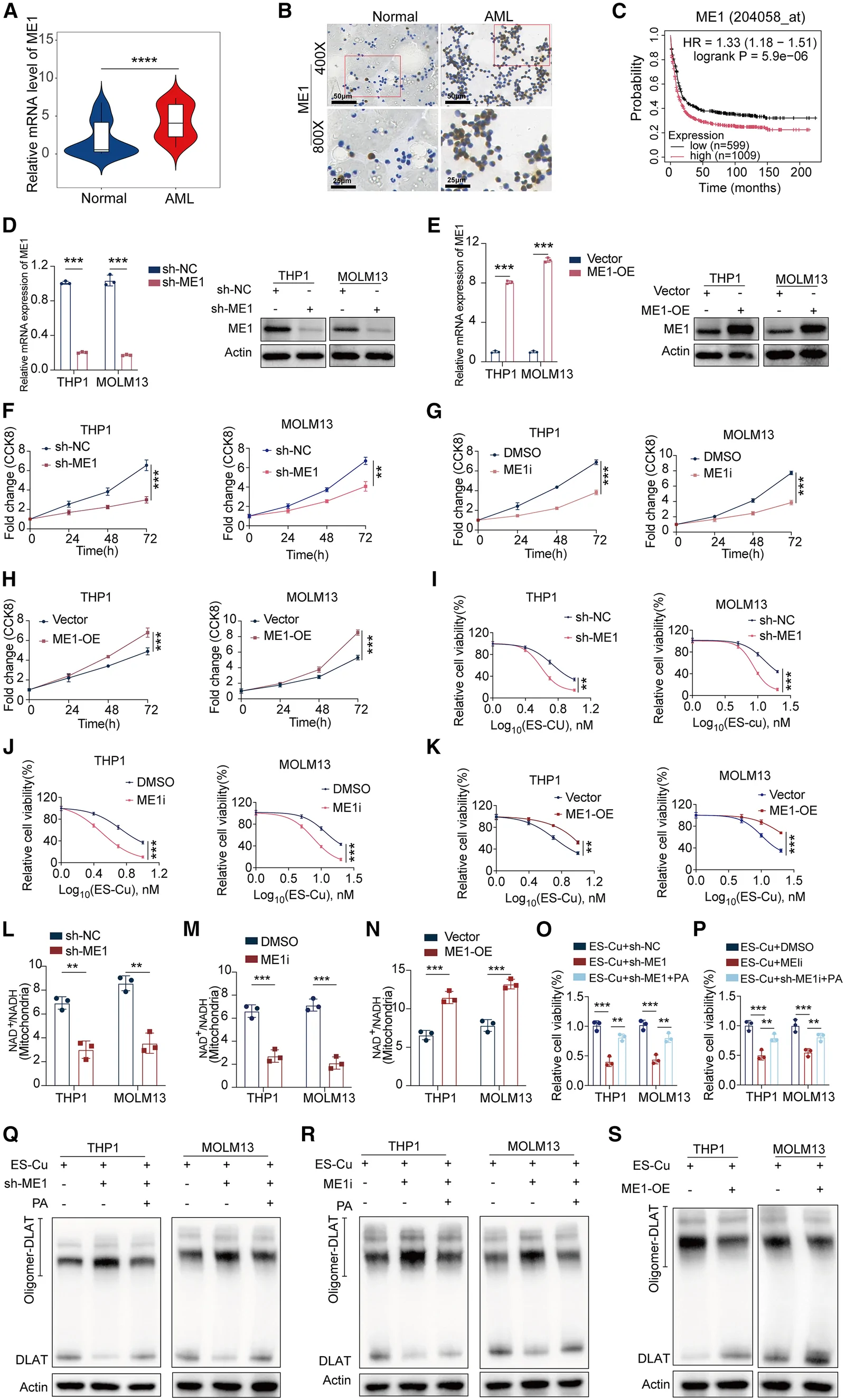
ME1 promotes resistance to cuproptosis by disrupting mitochondrial NAD^+^/NADH homeostasis (A) Comparison of ME1 mRNA expression in PBMCs from patients with AML and healthy controls (AML: *n* = 55, normal: *n* = 40, biologically independent samples). (B) Representative IHC images show ME1 protein expression in bone marrow (BM) from primary AML patients and normal BM. Scale bars: 50 μm (upper), 25 μm (lower). (C) OS analysis of the effect of ME1 expression patterns in the AML patients using Kaplan-Meier Plotter database. (D and E) ME1 mRNA and protein levels in stable ME1-knockdown and ME1-overexpression cells were detected by RT-qPCR and western blot, respectively. (F–H) Cellular viability was quantitatively assessed using CCK-8 assays at specified time points (0, 24, 48, and 72 h) following stable ME1 knockdown (F), treatment with 50 μM ME1 inhibitor (G) and ME1-overexpression (H) in THP1 and MOLM13 cells. (I–K) THP1 and MOLM13 cells with ME1-knockdown (I), treated with 50 μM ME1 inhibitor (J) and ME1-overexpression (K) were exposed to various concentrations of ES-Cu (ratio 1:1), cell viability was assessed using the CCK-8 assay. (L–N) Mitochondrial NAD^+^/NADH ratios in THP1 and MOLM13 cells after ME1 knockdown (L), pharmacological ME1 inhibition with ME1i (50 μM) (M), or ME1 overexpression (N). (O and P) ES-Cu-induced cell death was assessed by CCK-8 assay. Pyruvate (PA) (1 mM) rescue experiments were performed in ME1-knockdown AML cells (O) or ME1i-treated AML cells (P) under ES-Cu exposure. (Q–S) Representative western blots of DLAT oligomerization in THP1 and MOLM13 cells exposed to ES-Cu. Pyruvate (1 mM) supplementation reduced DLAT oligomerization induced by ME1 knockdown (Q) or ME1i treatment (R), whereas ME1 overexpression decreased DLAT oligomerization under ES-Cu treatment (S). Data are presented as mean ± SD from three independent experiments. Student’s *t* test was used in (A, D, E, and L–N), two-way ANOVA was used in (F–K) and one-way ANOVA was used (O and P). ∗∗*p* < 0.01, and ∗∗∗*p* < 0.001 vs. control.

Recent studies suggest that ME1 affects the mitochondrial NAD^+^/NADH homeostasis through the malate-aspartate shuttle (MAS).^40^ The delipoylation of lipoylated DLAT (lip-DLAT) is dependent on NAD^+^.^41^^,^^42^ Cu binding to lipoylated DLAT induces its oligomerization, thereby triggering cuproptosis.^12^ Based on these findings, we hypothesize that ME1 deficiency inhibits delipoylation of lip-DLAT by reducing NAD^+^, leading to the accumulation of lipoylated DLAT. Our results show that inhibition of ME1 leads to a decrease in mitochondrial NAD^+^/NADH levels (Figures 6L and 6M), accompanied by an increase in DLAT oligomerization (Figure 6Q). Similarly, treatment with the ME1 inhibitor promoted DLAT oligomerization (Figure 6R). To test whether NAD^+^/NADH imbalance mediates the effect of ME1 inhibition on cuproptosis sensitivity, we supplemented cells with pyruvate, a redox-active metabolite that supports NAD^+^ regeneration.^43^ In both ME1-knockdown and ME1 inhibitor-treated AML cells, pyruvate reduced DLAT oligomerization triggered by ME1 suppression under ES-Cu treatment (Figures 6Q and 6R). Consistently, pyruvate restored cell viability under ES-Cu treatment (Figures 6O and 6P). However, overexpression of ME1 elevated the mitochondrial NAD^+^/NADH ratio (Figure 6N) accompanied by a concurrent decrease in the levels of oligomer-DLAT (Figure 6S). These results suggest that ME1 inhibition disrupts mitochondrial NAD^+^/NADH homeostasis, thereby promoting DLAT oligomerization and sensitizing AML cells to cuproptosis.

### Combination of ME1 inhibitor with copper ionophore effectively inhibits AML progression

FDX1 suppression significantly increased ME1 expression in AML cells. To determine whether ME1 mediates FDX1-dependent regulation of cuproptosis, we knocked down ME1 in cells with stable FDX1 knockdown (Figures 7A and 7B). ME1 knockdown restored DLAT oligomerization that had been reduced by FDX1 deficiency under ES-Cu treatment (Figure 7C) and reversed FDX1-deficiency-induced cuproptosis resistance (Figures 7D and 7E). These findings suggested that FDX1 promotes cuproptosis through the downregulation of ME1, which regulated lipoylated DLAT.

**Figure 7:**
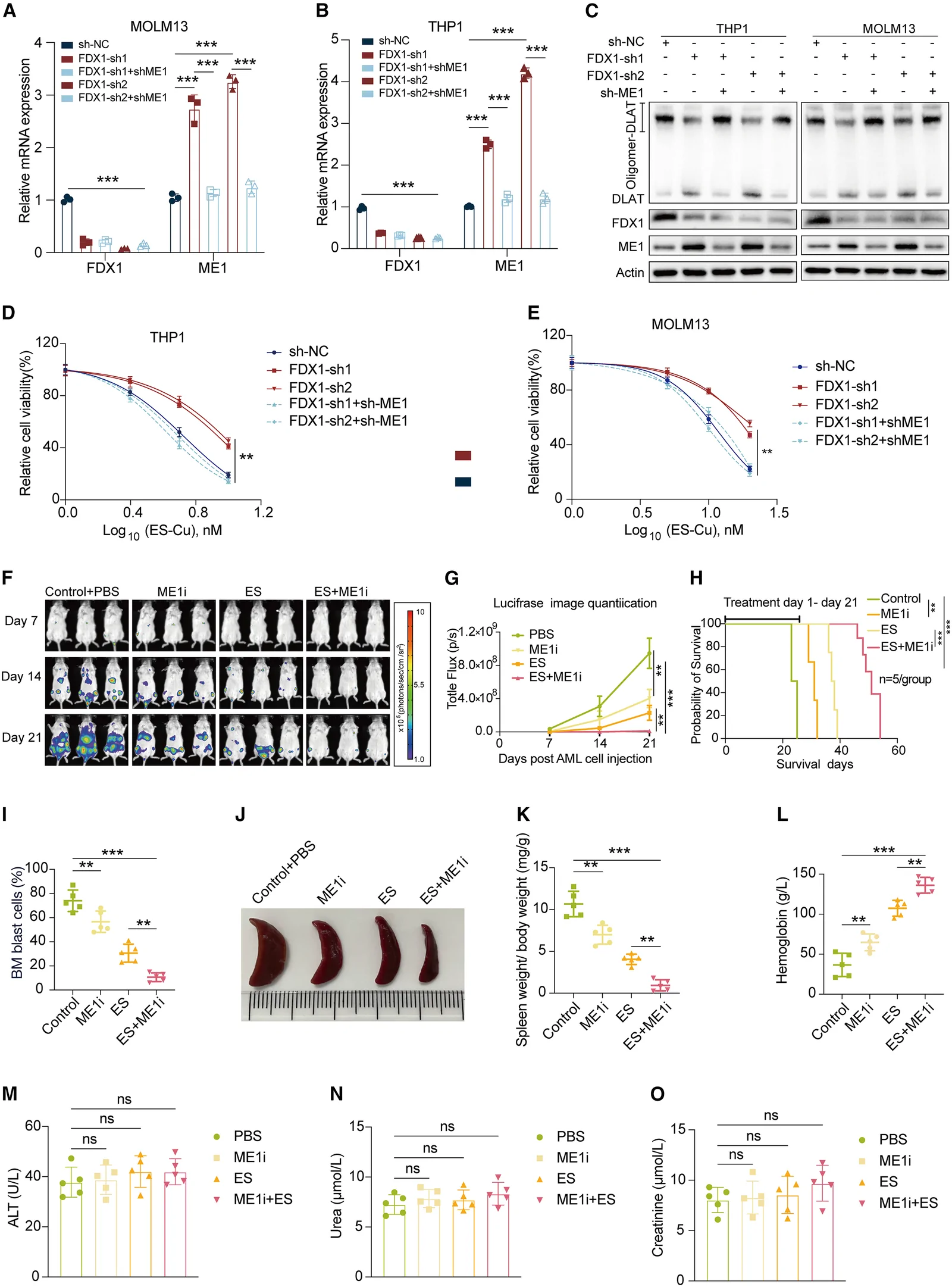
Combination of ME1 inhibitor with copper ionophore effectively inhibits AML progression (A and B) RT-qPCR analysis of FDX1 and ME1 mRNA levels in FDX1-knockdown and FDX1/ME1-double-knockdown AML cells. (C) Western blot analysis of DLAT oligomerization induced by ES-Cu (5 nM for THP1 cells; 10 nM for MOLM13 cells) in FDX1-knockdown and FDX1/ME1-double-knockdown AML cells. (D and E) CCK-8 assays quantifying ES-Cu-mediated cell viability in FDX1-knockdown and FDX1/ME1-double-knockdown AML cells. (F and G) Tumor growth was monitored weekly by bioluminescence imaging in MOLM13-engrafted NSG mice (F) and the quantification of luciferase signals for all mice per group (G). (H) Kaplan-Meier analysis of survival of MOLM13-engrafted NSG mice treated with ES, ME1 inhibitor, or a combination of ES and ME1 inhibitors was assessed. (I) Percentage of blasts in bone marrow in leukemic NSG mice after 3 weeks treatment. (J–L) Representative spleen images (J), spleen weight (K), and hemoglobin levels (L) in leukemic NSG mice after 3 weeks treatment. (M and O) The histogram presents serum levels of ALT, urea, and creatinine for each experimental group, following treatment with or without ES and the ME1 inhibitor. Data are presented as mean ± SD from three independent experiments or five mice per group. One-way ANOVA was used in (A, B, G, I, and K–O), two-way ANOVA was used in (D and E), and survival curves were estimated using the Kaplan-Meier method and compared using a two-sided log rank test in (H). ∗∗*p* < 0.01, and ∗∗∗*p* < 0.001 vs. control; ns, not significant.

To assess the potential of cuproptosis as a therapeutic strategy for AML, an *in vivo* study was conducted utilizing a combination of copper ionophore and ME1 inhibitors. MOLM13 cells were injected into the tail vein of NSG mice of both sexes. ME1 inhibitor monotherapy reduced leukemia burden compared with vehicle treatment. Notably, combined treatment with elesclomol and the ME1 inhibitor further reduced leukemia burden compared with either monotherapy (Figures 7F and 7G). Furthermore, this combined therapeutic approach significantly prolonged survival (Figure 7H), decreased the percentages of leukemic blasts in bone marrow (Figure 7I), prevented splenomegaly (Figures 7J and 7K), and alleviated anemia (Figure 7L) compared to either monotherapy. The assessment of the safety and toxicity of elesclomol and ME1 inhibitors is a pivotal consideration in clinical applications. Blood biochemical analyses showed no statistically significant differences in ALT, urea, and creatinine levels post-medication, suggesting no obvious hepatic or renal toxicity in mice treated with elesclomol, ME1 inhibitor, or their combination (Figures 7M and 7O). In summary, these findings demonstrated that the combination of ME1 inhibitor and elesclomol effectively reverses cuproptosis resistance in AML, representing a promising therapeutic strategy for AML.

## Discussion

Copper homeostasis dysregulation has emerged as a critical driver of tumorigenesis, with cancer cells exhibiting elevated copper uptake to sustain their metabolic and proliferative demands.^44^^,^^45^^,^^46^ Elevated serum copper concentration in AML patients is significantly associated with adverse clinical outcomes, including reduced complete remission (CR) rates and poorer long-term survival,^11^ which is consistent with our findings. These findings suggest copper may serve as both a prognostic biomarker and a therapeutic target in AML. Recent advances have identified cuproptosis, a copper-dependent form of mitochondrial cell death,^12^ as a potential therapeutic target. A recent study showed that inhibition of heme biosynthesis triggers copper-dependent cell death through mitochondrial dysfunction and dysregulation of copper handling.^20^ This study supports the concept that cuproptosis can be therapeutically exploited in AML. Their work mainly focused on heme biosynthesis as an upstream metabolic vulnerability, whereas our study starts from the clinical observation of elevated serum copper levels in AML patients and demonstrates that reduced FDX1 expression in AML promotes both cell proliferation and cuproptosis resistance. Mechanistically, FDX1 upregulation activates the ROS/p53/ME1 axis by promoting mitochondrial damage. ME1 modulates cuproptosis sensitivity by regulating mitochondrial NAD^+^/NADH homeostasis. Notably, the combination of the ME1 inhibitor and the copper ionophore (such as elesclomol) synergistically inhibits AML progression both *in vitro* and *in vivo*.

FDX1 is a mitochondrial iron-sulfur protein that participates in electron transport. FDX1 demonstrates significantly reduced expression in hepatocellular carcinoma (HCC) tissues, exhibiting negative correlations with microvascular invasion (MVI) and advanced TNM staging, indicative of its potential tumor-suppressive function. FDX1 downregulation promotes mitophagy and tumor progression through PI3K/AKT pathway activation. Furthermore, FDX1 deficiency induces dual metabolic dysregulation in lipid metabolism and amino acid metabolism in HCC cells, thereby sustaining tumor cell proliferation.^47^ In thyroid carcinoma, FDX1 expression is markedly diminished in malignant tissues compared to non-neoplastic counterparts, with copper ionophore effectively suppressing malignant progression through cuproptosis induction in thyroid cancer cells.^48^ Recent investigations in triple-negative breast cancer reveal that AKT1 phosphorylates FDX1 to promote cuproptosis resistance.^49^ FDX1 exhibits significant transcriptional downregulation in clear cell renal cell carcinoma (ccRCC), correlating with adverse clinical outcomes as an independent prognostic indicator. Functional analyses demonstrate that FDX1 overexpression suppresses ccRCC tumorigenesis and metastatic dissemination through FMR1-mediated pathway activation.^50^ Our study identifies FDX1 as the most significantly downregulated CRG in AML patients, low FDX1 expression was related to worse OS in AML patients. FDX1 deficiency promotes AML cell proliferation and confers resistance to cuproptosis, whereas FDX1 overexpression inhibits AML progression and enhances the sensitivity of cuproptosis.

FDX1 overexpression exacerbates mitochondrial damage, triggering ROS accumulation and subsequent p53 activation, these findings align with prior reports linking ROS and p53 activation.^24^^,^^51^^,^^52^^,^^53^ p53 is involved in the transcriptional regulation of a multitude of target genes involved in cellular processes such as cell cycle progression, senescence, cell death, and metabolic regulation. In particular, cuproptosis is intricately linked to cellular metabolic pathways. In our study, p53 transcriptionally represses ME1 expression, and thereby modulates pyruvate metabolism. These observations suggest that FDX1 upregulation activates a ROS/p53/ME1 signaling axis by disrupting mitochondrial function, thereby influencing the metabolism of AML cells.

ME1 is a cytosolic malate enzyme that catalyzes the conversion of malate to pyruvate, processes that are indispensable for tumor metabolism and redox regulation.^37^^,^^54^^,^^55^^,^^56^ Zhang et al. showed that ME1 is highly expressed in AML and predicts poor prognosis, potentially through activation of the IL-6/JAK2/STAT3 pathway.^27^ Hu et al. further demonstrated that NRF2 maintains redox balance through ME1 in NPM1-mutated AML and that NRF2 inhibition synergizes with venetoclax.^28^ In agreement with these reports, we observed elevated ME1 expression in AML and an association between high ME1 expression and poor prognosis. ME1 links glycolysis to the TCA cycle by recycling malate to produce pyruvate as the central mediator of the entire cellular metabolic network and serves as a bridge between cytosolic and mitochondrial metabolism.^57^ However, the role of ME1 in cuproptosis regulation was previously undefined. ME1 deficiency significantly inhibits AML cell proliferation and enhances sensitivity to cuproptosis. Cuproptosis refers to a mitochondrial cell death pathway, copper directly binds and promotes the oligomerization of lip-DLAT,^12^ and lipo-DLAT delipoylation is dependent on NAD^+^,^41^^,^^42^ ME1 inhibition decreases the mitochondrial NAD^+^/NADH ratio by regulating the MAS cycle.^40^ Consistent with previous research, our results showed that both ME1 deficiency and ME1 inhibitor reduced the mitochondrial NAD+ level, accompanied by an increase in lip-DLAT and oligomer-DLAT levels, under ES-Cu treatment. Our work reveals that ME1 inhibition or depletion enhances cuproptosis sensitivity by disrupting DLAT delipoylation, a process dependent on NAD^+^ availability.

The elevated basal copper levels in AML patients provide a therapeutic window for cuproptosis induction. However, FDX1 downregulation in AML patients poses a significant barrier. Our combinatorial strategy of elesclomol and ME1 inhibitors overcomes this resistance by simultaneously leveraging AML’s inherent copper overload and ME1 overexpression. Blood biochemical analyses further indicated that the administration of ES-Cu did not elicit liver or kidney damage in mice. Both *in vitro* and *in vivo* experimental results suggest that a combination therapy targeting ME1 inhibition and the induction of cuproptosis may constitute an effective and safe therapeutic strategy for the treatment of AML patients. Nevertheless, because key mechanistic markers were not directly assessed in tumor cells isolated from the *in vivo* treatment group, direct *in vivo* validation of the proposed mechanistic axis remains an important limitation and warrants further investigation in future studies with sufficient animal-derived materials.

Collectively, our findings indicate that reduced FDX1 expression promotes AML cell proliferation and resistance to cuproptosis, whereas FDX1 overexpression enhances AML cell sensitivity to cuproptosis through activation of the ROS/p53/ME1 axis. ME1 inhibition disrupts mitochondrial NAD^+^/NADH homeostasis, promotes DLAT oligomerization, and rescues FDX1 deficiency-induced resistance to cuproptosis. These findings suggest that leveraging elevated copper availability and targeting ME1 may represent a potential therapeutic strategy for AML.

### Limitations of the study

Several limitations of this study should be acknowledged. First, the broader applicability of ME1 inhibition in cancers with copper dysregulation remains to be tested. Second, further pharmacokinetic and safety optimization of copper ionophore and ME1 inhibitor combinations will be required before clinical translation. In addition, although serum copper, FDX1, and ME1 were individually associated with AML progression and/or prognosis, complete paired data for these three variables together with survival information were not available for all patients. Consequently, we were unable to reliably assess their potential interaction or construct a three-variable prognostic model in the present cohort. Future prospective multicenter studies with complete matched clinical and molecular data are needed to validate the prognostic value and therapeutic relevance of the copper-FDX1-ME1 axis.

## Resource availability

### Lead contact

Requests for further information and resources should be directed to and will be fulfilled by the lead contact, Xiaozhong Wang (wangxiaozhong@ncu.edu.cn).

### Materials availability

This study did not generate new, unique reagents.

### Data and code availability

• The RNA-sequencing data generated in this study have been deposited in the NCBI Gene Expression Omnibus (GEO) and are publicly available under accession number GSE343704.
• This study does not report original code.
• Additional information required to reanalyze the data reported in this study is available from the lead contact upon reasonable request.

## Acknowledgments

This study was supported by the 10.13039/501100001809National Natural Science Foundation of China (grant no. 82160405).

## Author contributions

X.W., B.H., and L.Y. designed the study. Y.X., J.Z., J.L., and F.Y. provided and analyzed patient clinical samples. L.Y., F.Z., B.Q., H.Z., Y.Q., and K.D. performed the experiments, analyzed the data, and prepared the figures and tables. L.Y. wrote the manuscript with assistance from X.W.

## Declaration of interests

The authors declare no competing interests.

## STAR★Methods

### Key resources table

**Table:** 

REAGENT or RESOURCE	SOURCE	IDENTIFIER
**Antibodies**		

FDX1 antibody	Abmart	Cat# T510671
DLAT antibody	Cell Signaling Technology	Cat# 12362
Lipoic Acid antibody	Abcam	Cat# ab58724
p53 antibody	Cell Signaling Technology	Cat# 9282
ME1 antibody	Abmart	Cat# TD4217
γ-H2AX antibody	Abmart	Cat# M63324
β-actin antibody	Proteintech	Cat# 66009-1-Ig

**Bacterial and virus strains**		

Lentiviral vector for FDX1 knockdown	Genechem	N/A
Lentiviral vector for FDX1 overexpression	Genechem	N/A
Lentiviral vector for ME1 knockdown	Genechem	N/A
Lentiviral vector for ME1 overexpression	Genechem	N/A
Lentiviral vector for p53 knockdown	Genechem	N/A
Luciferase expression vector	Genechem	N/A

**Biological samples**		

Peripheral blood and bone marrow samples from patients with AML	The Second Affiliated Hospital of Nanchang University	This paper
Peripheral blood and bone marrow samples from healthy controls	The Second Affiliated Hospital of Nanchang University	This paper

**Chemicals, peptides, and recombinant proteins**		

Elesclomol (ES)	Selleck Chemicals	Cat# S1052
Copper(II) chloride (CuCl_2_)	Sigma-Aldrich	Cat# 751944
Tetrathiomolybdate (TTM)	Sigma-Aldrich	Cat# 323446
ME1 enzymatic inhibitor (ME1i)	MedChemExpress	Cat# HY-124861
N-acetyl-L-cysteine (NAC)	MedChemExpress	Cat# HY-B0215
CellROX Deep Red reagent	Invitrogen	Cat# C10491
Ferrostatin-1 (Fer-1)	Sigma-Aldrich	Cat# ST9H93D8E91A
Z-VAD-FMK	Sigma-Aldrich	Cat# ST9H9BC12E93
BOC-D-FMK	Sigma-Aldrich	Cat# ST9H9BC16AB7
Hydrogen peroxide (H_2_O_2_)	Sigma-Aldrich	Cat# H1009
Pyruvate	Sigma-Aldrich	Cat# P2256
RPMI 1640 medium	Gibco	Cat#C11875500BT
Fetal bovine serum	Fuheng Biology	Cat# FH100-900

**Critical commercial assays**		

Copper (Cu) Assay Kit, PAESA method	Autobio Diagnostics	Cat# 6840–411
BCA Protein Assay Kit	Beyotime	Cat# P0011
SDS-PAGE Kit	Epizyme Biotech	Cat# PG610
Western blot stripping buffer	Servicebio	Cat# G2078
Reverse transcription/qPCR reagents	Takara	Cat# RR092
Cell Counting Kit-8 (CCK-8)	APExBIO	Cat# K1018
Annexin V-FITC/PI staining kit	BioLegend	Cat# 640932
Propidium Iodide Solution	BioLegend	Cat# 421301
JC-1 mitochondrial membrane potential assay	Beyotime	Cat# c2003S
Enzymatic ChIP Assay Kit	Beyotime	Cat# P2083S
Mitochondria Isolation Kit	Beyotime	Cat# C3602S
NAD^+^/NADH Assay Kit	Beyotime	Cat# S0176S

**Deposited data**		

RNA-seq data from FDX1-knockdown and control MOLM13 cells	NCBI Gene Expression Omnibus (GEO)	GEO: GSE343704
p53 ChIP-seq dataset	NCBI Gene Expression Omnibus (GEO)	GEO: GSM2501568
TCGA AML gene-expression and clinical data	The Cancer Genome Atlas	TCGA-LAML

**Experimental models: Cell lines**		

MOLM13 human AML cells	Fuheng Biology	FH1018
THP1 human AML cells	ATCC	TIB-202
NB4 human AML cells	Fuheng Biology	FH0115
HL60 human AML cells	ATCC	CCL-240
U937 human leukemia cells	ATCC	CRL-1593.2

**Experimental models: Organisms/strains**		

NSG mice, 5–6 weeks, both sexes	GemPharmatech	T001475

**Oligonucleotides**		

qRT-PCR primers used in this study	Sangon Biotech	Sequences listed in Table S1
FDX1 and ME1 knockdown targeting sequences	Genechem	Sequences listed in Table S1
ME1 promoter ChIP-qPCR primers	Sangon Biotech	Sequences listed in Table S1

**Software and algorithms**		

GraphPad Prism	GraphPad Software	Version 10.0
R	R Foundation for Statistical Computing	Version 3.6.3
Gene Set Enrichment Analysis (GSEA)	Broad Institute	GSEA
DAVID	NIH	DAVID
UCSC Xena	UCSC	Xena Browser
GEPIA	Tang et al.	GEPIA
DepMap/CCLE	Broad Institute	DepMap
JASPAR	JASPAR database	JASPAR
GEO	NCBI	Gene Expression Omnibus
TCGA	National Cancer Institute	TCGA-LAML

**Other**		

Automated biochemical analyzer	Mindray	BS-2800M
Transmission electron microscope	Hitachi	H-7650
Bioluminescence imaging system	Berthold technologies	LB983
Real-time PCR system	Applied biosystems	QuantStudio^TM^ 5
Flow cytometer	Agilent Flow Cytometers	NovoCyte Quanteon
Confocal microscope	Nikon	C2

### Experiment model and study participant details

#### Human participants

Peripheral blood and bone marrow samples were obtained from 126 patients with AML and 76 healthy controls at the Second Affiliated Hospital of Nanchang University. AML was diagnosed according to established clinical and hematological criteria. The study was approved by the Medical Ethics Committee of the Second Affiliated Hospital of Nanchang University. The requirement for informed consent was waived because of the retrospective study design and the use of archived, de-identified clinical specimens and data. Demographic information, including age and sex, was collected from the clinical records. No statistically significant differences in age or sex distribution were observed between the analyzed groups, and age and sex did not show a statistically significant association with the main results in our cohort.

#### Cell lines

Human AML cell lines THP1, HL60 and U937 were obtained from the American Type Culture Collection. MOLM13 and NB4 were obtained from the FUHENG Biology. Cells were cultured in RPMI 1640 medium supplemented with 10% fetal bovine serum at 37°C in a humidified atmosphere containing 5% CO_2_. All cell lines were authenticated by short tandem repeat (STR) profiling and routinely screened for mycoplasma contamination, with all tests yielding negative results.

#### Mouse models

NSG mice, 5–6 weeks of age, were obtained from GemPharmatech. Both male and female mice were used in the study. Luciferase-expressing MOLM13 cells (5 × 10^5^ cells per mouse) were injected through the tail vein to establish the AML xenograft model. After successful engraftment was confirmed by bioluminescence imaging, mice were assigned to the indicated experimental groups and treated with PBS, elesclomol (10 mg/kg), ME1 inhibitor (50 mg/kg), or their combination three times per week. Leukemia progression was monitored by serial bioluminescence imaging, and mouse survival was recorded. Bone marrow cells were examined by Wright–Giemsa staining. Hemoglobin level of peripheral blood (PB) was measured by automated Hematology Analyzer. Additionally, levels of ALT, urea, and creatinine were measured using a Mindray analyzer. All animal procedures were approved by the Animal Care Committee of Nanchang University (approval no. NCULAE-20221031186) and performed in accordance with institutional guidelines and applicable regulations governing animal research. The animal experiments are reported in accordance with the ARRIVE guidelines.

### Method details

#### Serum copper level

Fresh peripheral blood samples were collected from AML patients and healthy controls using serum collection tubes. After clotting, samples were centrifuged at 4,000 rpm to separate the serum. Serum copper concentrations were measured using a commercially available Copper (Cu) Assay Kit based on the PAESA method (Autobio Diagnostics Co., Ltd., Zhengzhou, China) according to the manufacturer’s protocol. The assay was based on the reaction between copper ions and the chromogenic reagent 3,5-DiBr-PAESA under acidic conditions, forming a colored complex whose absorbance is proportional to the copper concentration. Briefly, 200 μL of Reagent 1 was added to each reaction well/tube, followed by 12 μL of deionized water for the blank, 12 μL of copper calibrator for the calibration reaction, or 12 μL of serum sample for the test reaction. After mixing, the reaction was incubated at 37°C for 5 min, and the initial absorbance value was recorded as A1. Subsequently, 50 μL of Reagent 2 was added, followed by incubation at 37°C for another 5 min. The final absorbance value was recorded as A2 at a main wavelength of 570 nm using Mindray automated biochemical analyzer (BS-2800M). The change in absorbance was calculated as ΔA = A2 − A1. Serum copper concentration was calculated according to the following formula:

Cuconcentration=ΔAsample/ΔAcalibrator×calibratorconcentration

A calibration curve was generated before sample measurement using a blank and copper calibrator (Figure S1). Commercial quality-control materials were analyzed in each run, and results were accepted only when control values fell within the laboratory’s predefined limits. All serum samples were measured under the same reaction conditions.

#### Reagents

Elesclomol (ES) (S1052, Selleck, USA), CuCl_2_ (751944, Sigma-Aldrich, USA), tetrathiomolybdate (TTM, 323446, Sigma-Aldrich, USA), ME1 enzymatic inhibitor (ME1i) (HY-124861, MCE, NJ, USA). N-acetylcysteine (NAC, HY-B0215, MCE, USA).

#### Western blotting (WB) and qRT-PCR

Cells were lysed using RIPA lysis buffer, which was supplemented with phosphatase and protease inhibitors (Beyotime), and protein concentrations were quantified employing a BCA Protein Assay Kit (Beyotime). The western blotting (WB) analysis utilized antibodies specific to FDX1 (T510671, Abmart), DLAT (#12362, CST), Lipoic Acid (ab58724, Abcam), p53 (#9282, CST), ME1 (TD4217, Abmart), γ-H2AX (M63324, Abmart), with β-actin (66009-1-Ig, Proteintech) as a loading control. Equal amounts of protein were separated by SDS-PAGE kit (Epizyme Biotech), transferred to PVDF membranes, and incubated with the indicated primary and HRP-conjugated secondary antibodies. Signals were visualized using enhanced chemiluminescence. After detection of the first target protein, the membranes were stripped with Western blot stripping buffer (Servicebio, G2078) for 10 min at room temperature, washed with TBST, reblocked, and incubated with primary antibodies against the other target proteins, followed by the corresponding secondary antibodies.

Total RNA was extracted using an RNA isolation kit (Beyotime), and RNA concentrations were measured using a NanoDrop 2000 spectrophotometer. RNA was reverse-transcribed into cDNA using a reverse transcription kit (Takara, Japan). Quantitative PCR was performed using SYBR Green qPCR reagents (Takara, Japan) on a Bio-Rad real-time PCR system. The cycling conditions consisted of an initial denaturation at 95°C for 30 s, followed by 40 cycles of 95°C for 5 s and 60°C for 30 s. Gene expression levels were calculated from the Ct values obtained by qPCR. All primers were synthesized by Sangon Biotech (Shanghai, China, Table S1).

#### Immunohistochemical staining (IHC) and hematoxylin-eosin (HE) staining

Bone marrow smears underwent effective blocking of endogenous peroxidase activity, followed by a series of incubations with the primary antibody, reaction enhancer, and enhanced enzyme-labeled sheep anti-mouse/rabbit IgG polymer. Color development was achieved through the application of DAB, with subsequent nuclear staining accomplished using hematoxylin.

Paraffin-embedded sections of liver and spleen tissues, harvested from animals, were sectioned to a uniform thickness of 4 μm and subjected to HE staining procedures. The stained sections were examined and photographed under a high-powered microscope.

#### Plasmids and transfection

Lentiviral vectors harboring knockdown and overexpression constructs for FDX1 or ME1 were sourced from Genechem (Shanghai, China). Stably transduced cell lines were generated by lentiviral infection, followed by rigorous selection using puromycin (5 μg/mL) or neomycin (600 μg/mL). The targeting sequences employed in these constructs are detailed in Table S1. Transduction efficiency was validated through qPCR and WB.

#### Cell viability

Cells were seeded in 96-well plates at a density of 1 × 10^4^ cells/well and treated with ES-Cu and/or additional reagents for 48 h. Subsequently, 10 μL of CCK-8 solution (APExBIO, USA) was added to each well and gently mixed. The plates were then incubated at 37°C in a humidified incubator with 5% CO_2_ for 2 h. Absorbance was measured at 450 nm using a microplate reader. The resulting absorbance values were used to assess cell viability and perform statistical analyses.

#### Flow cytometry

Cell death was assessed using an Annexin V/PI staining kit (Biolegend, USA). AML cells were collected by centrifugation at 2,000 rpm for 5 min, washed twice with cold PBS, and resuspended in 100 μL Annexin V binding buffer. Cells were stained with 5 μL APC-conjugated Annexin V and 5 μL propidium iodide solution for 15 min at room temperature in the dark. Following centrifugation, cells were resuspended in 500 μL Annexin V binding buffer and analyzed by Agilent flow cytometry.

Cells were incubated in complete medium containing 5 μmol/L of CellROX Deep Red reagent (Invitrogen, Carlsbad, CA) at 37°C for 30 min to achieve staining. Subsequently, the cells underwent three washes with phosphate-buffered saline (PBS) and were fixed using 4% formaldehyde. Cellular ROS fluorescence was immediately measured using an Agilent flow cytometer.

Logarithmically growing cells were collected, washed twice with cold PBS, and fixed in 70% ethanol at −20°C for at least 2 h. After three PBS washes, cells were resuspended in 450 μL PBS and treated with 50 μL RNase (100 μg/mL, Beyotime, Shanghai, China) and stained with 10 μL of PI (Biolegend, USA) staining buffer at 37°C for 30 min in the dark. Agilent flow cytometer was used to analyze cell cycle progression. A total of 10,000 valid cellular events were acquired per sample, and the percentages of cells in the G0/G1, S, and G2/M phases were determined using NovoExpress software.

#### Transmission electron microscopy (TEM)

The cells were immobilized in a 2.5% glutaraldehyde solution at 4°C for 24 h. Following this, the specimens underwent additional fixation with a 1% osmic acid solution for 2 h. After dehydration, embedding, and sectioning, the samples were stained, visualized, and captured using a transmission electron microscope (TEM; Hitachi, H-7650, Japan).

#### Mitochondrial membrane potential

Cells were stained using a working solution of JC-1 (Beyotime, Shanghai, China) and subjected to incubation at 37°C for 20 min, then the cells were washed twice with a buffer specifically formulated for JC-1 staining. JC-1 aggregates and JC-1 monomers captured by confocal microscopy, and JC-1 aggregate fluorescence was quantified by flow cytometry.

#### RNA-sequencing and bioinformatics analysis

Total RNA was extracted using TRIzol reagent (Takara) and subsequently treated with DNase I. The resulting RNA libraries were sequenced on the Illumina sequencing platform (Illumina, San Diego, CA) at Oebiotech (Shanghai, China), generating 150 bp paired-end reads. Gene Set Enrichment Analysis (GSEA) was performed using the complete ranked gene list. Genes were ranked according to their differential expression statistics between groups, and enrichment of predefined gene sets was evaluated based on the global ranking of all detected genes. For conventional functional annotation of differentially expressed genes, genes meeting the predefined threshold of |logFC| > 1.5 were subjected to Gene Ontology (GO) and Kyoto Encyclopedia of Genes and Genomes (KEGG) pathway enrichment analyses using the DAVID database. Publicly available datasets were obtained from the GEO database and TCGA database. Single-sample GSEA (ssGSEA) was further performed in R version 3.6.3 to calculate pathway enrichment scores for individual samples.

#### Chromatin immunoprecipitation (ChIP)

ChIP was executed utilizing the Enzymatic ChIP Assay Kit (Beyotime, Shanghai, China). Cellular samples were subjected to crosslinking, lysis, and sonication processes. Chromatin fractions were immunoprecipitated with antibodies specific to p53 (#9282, CST) and IgG as controls. Following this, protein A/G beads were utilized for the isolation of protein-DNA complexes. The resultant purified DNA was subsequently analyzed through qPCR using primers as indicated in Table S1.

#### Mitochondrial isolation and NAD+/NADH measurement

Mitochondria were isolated from AML cells using a Mitochondrial Isolation Kit (Beyotime, Shanghai, China), NAD^+^ levels in isolated mitochondria were quantified using an NAD^+^/NADH assay kit (Beyotime, Shanghai, China), strictly following the manufacturer’s instructions.

### Quantification and statistical analysis

Statistical analyses were conducted using GraphPad Prism 10.0. Unless otherwise indicated, quantitative data are presented as mean ± SD. For *in vitro* experiments, n represents the number of biologically independent experiments, with at least three independent experiments performed. For human and animal studies, n represents the number of biologically independent participants or animals, respectively. Exact sample sizes and definitions of n are provided in the corresponding figure legends. To assess statistical significance, we employed the two-tailed Student’s *t* test for comparisons between two groups and one-way ANOVA followed by multiple comparison test for comparisons involving more than two groups. Survival analysis was performed using Kaplan–Meier curves. A *p*-value of less than 0.05 was considered statistically significant.

### Additional resources

Not applicable.
