# Supplementary material for: FDX1 suppresses acute myeloid leukemia progression and reverses cuproptosis resistance via the ROS/p53/ME1 axis
Source: iScience. 2026 Oct 26;29(11):117968. doi: 10.1016/j.isci.2026.117968 (PMC13734783; doi:10.1016/j.isci.2026.117968)
Supplement: Supplementary file 1 [file mmc1.pdf]

iScience, Volume *29*

Supplemental information

FDX1 suppresses acute myeloid leukemia progression and reverses cuproptosis resistance via the ROS/p53/ME1 axis

Lihua Yao, Fangmin Zhong, Qin Bai, Huan Zeng, Yuxiang Qiu, Fangyi Yao, Yanmei Xu, Jing Zhang, Jin Lin, Keyu Deng, Bo Huang, and Xiaozhong Wang

Document S1. Supplemental information, Figures S1 – S3, and Tables S1 – S3

Figure S1. Serum copper standard curve.

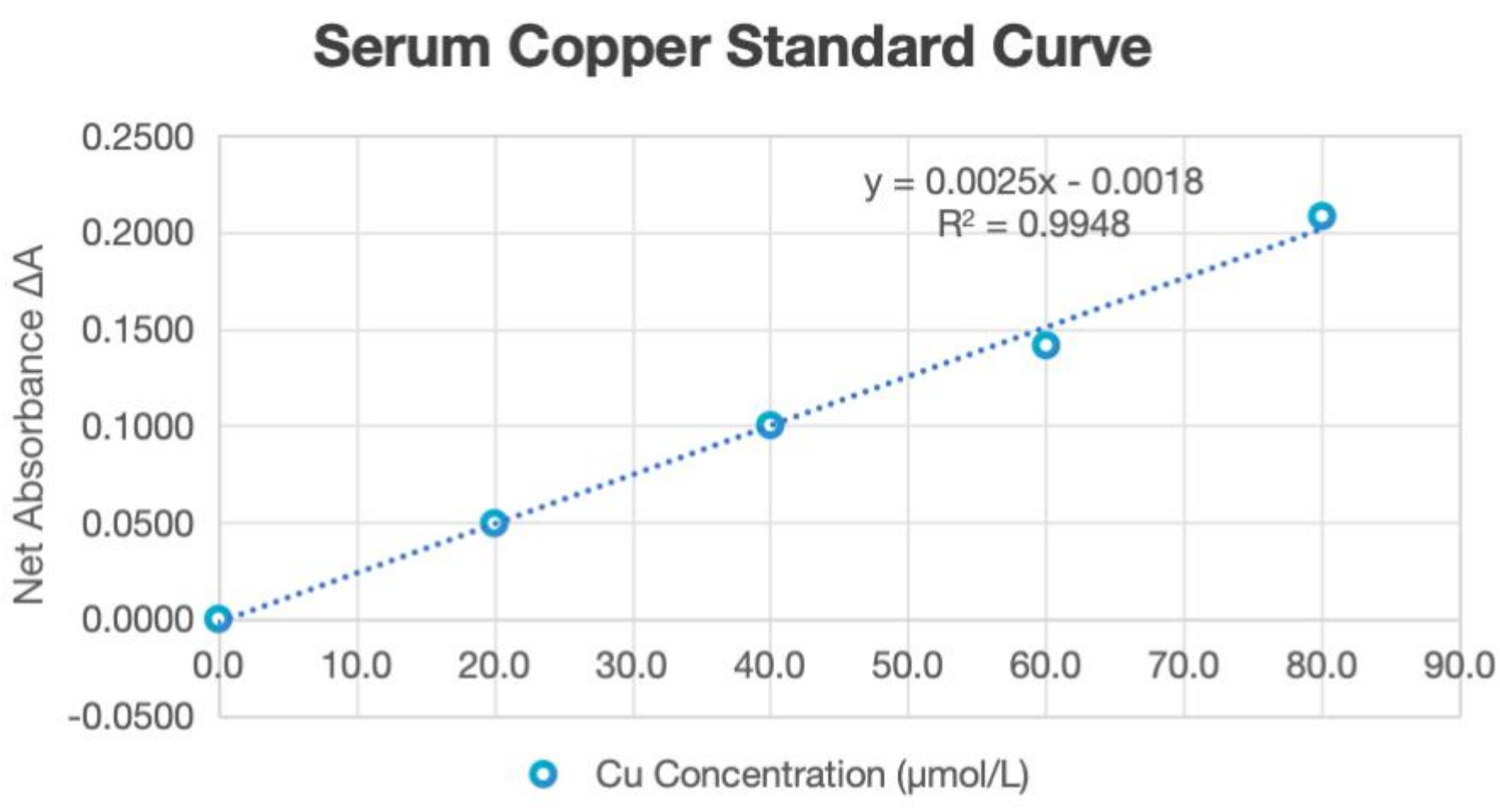

Figure S1. Serum copper standard curve.

The standard curve for serum copper measurement was generated using the PAESA method. Net absorbance values (ΔA) were calculated by subtracting the blank absorbance from the calibrator absorbance and plotted against copper concentration (μmol/L). A linear relationship was observed between copper concentration and net absorbance, with the regression equation y = 0.0025x - 0.0018, R2 = 0.9948, indicating excellent linearity within the calibration range.

Figure S2. The gene expression matrix for AML cell lines was obtained from the CCLE dataset.

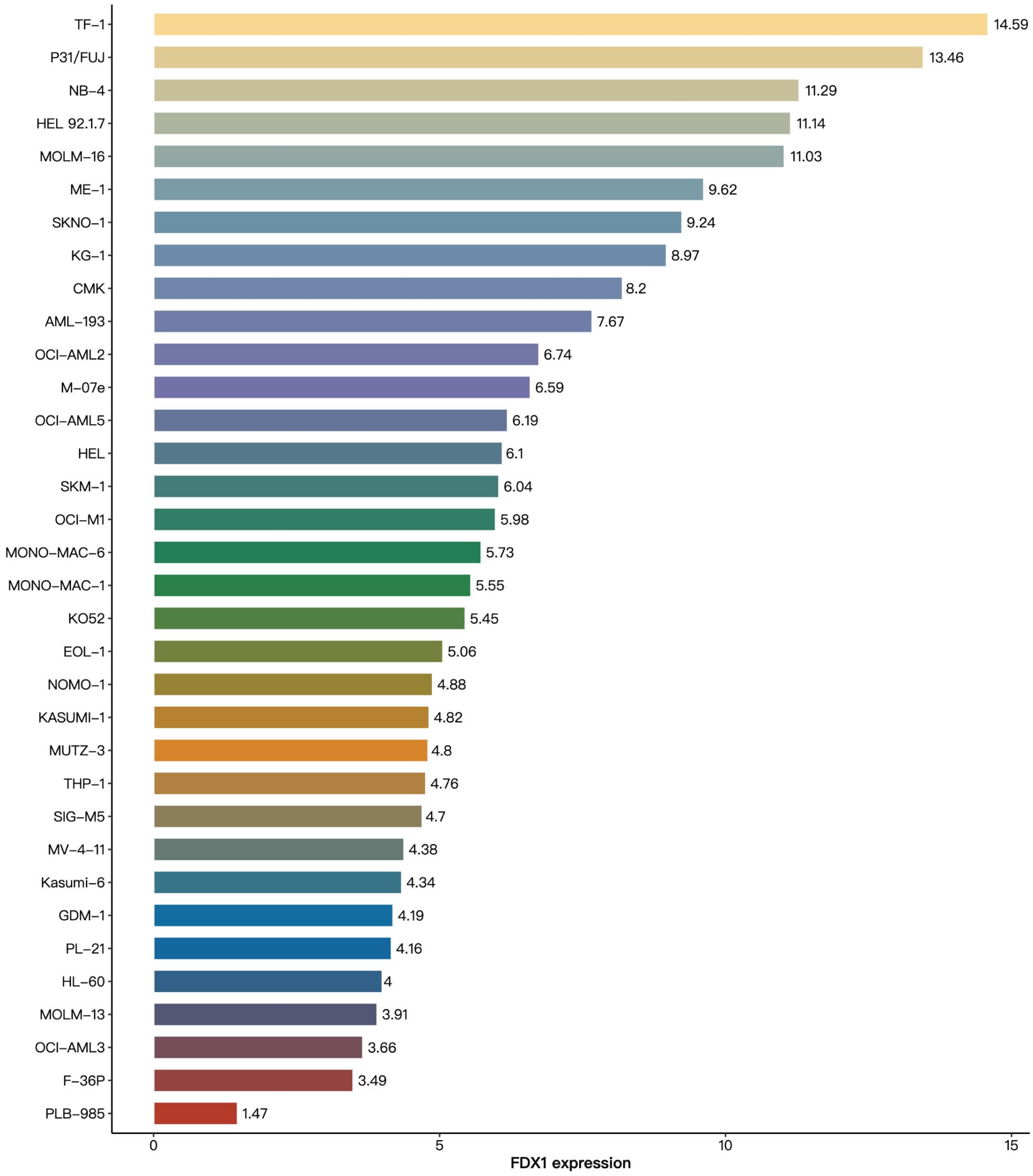

Figure S2. FDX1 expression across AML cell lines in the CCLE dataset. FDX1 expression data for the indicated AML cell lines were obtained from the Cancer Cell Line Encyclopedia (CCLE) dataset through the DepMap portal (https://depmap.org/portal/data_page/?tab=allData).

Figure S3. FDX1 overexpression enhances AML cell sensitivity to cuproptosis .

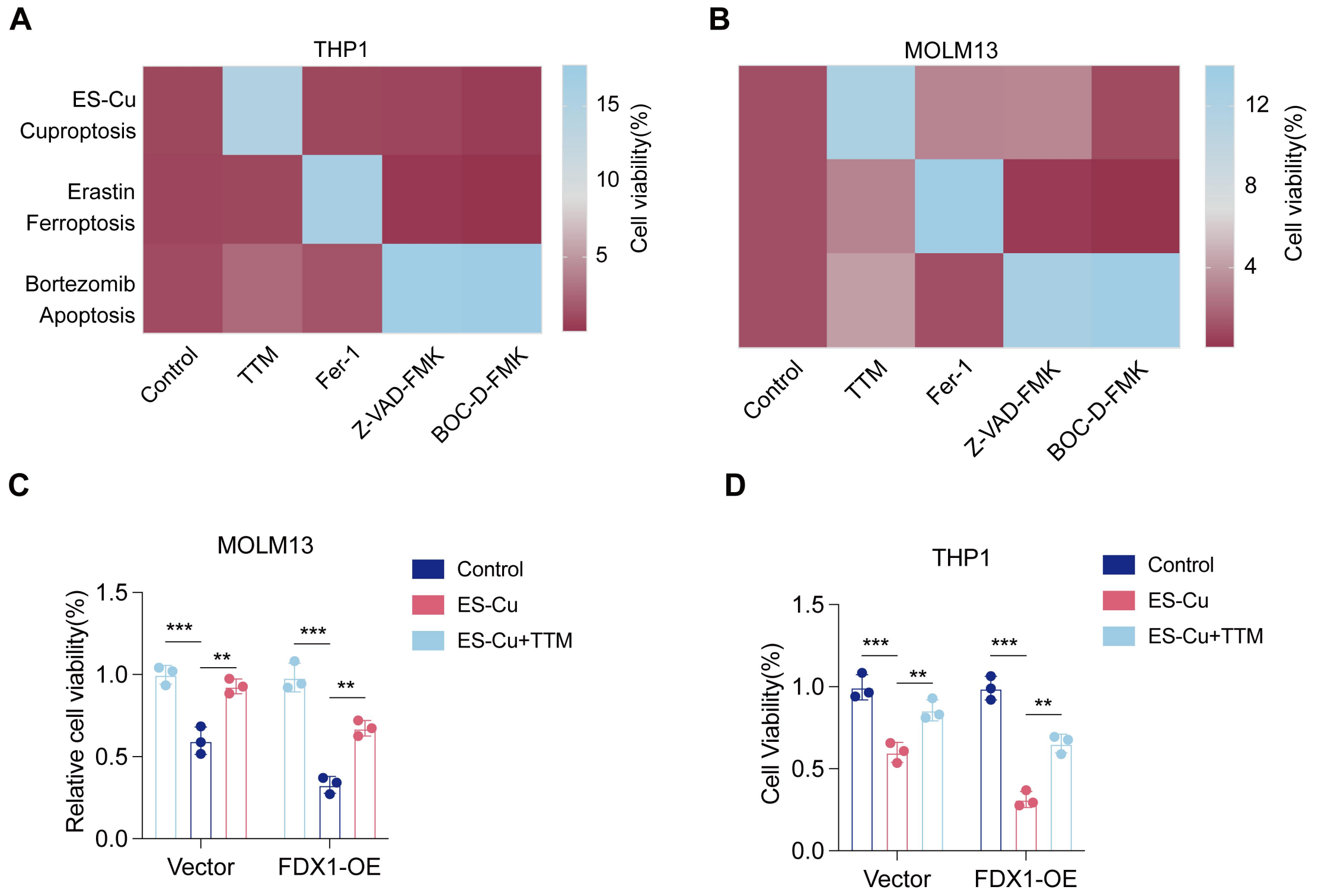

Figure S3. FDX1 overexpression enhances AML cell sensitivity to cuproptosis. A-B. Cell death specificity was assessed in THP1 and MOLM13 cells using representative inducers and inhibitors of cuproptosis, ferroptosis, and apoptosis. ES-Cu, erastin, and bortezomib were used to induce cuproptosis, ferroptosis, and apoptosis, respectively. TTM, Fer-1, Z-VAD-FMK, and BOC-D-FMK were used as inhibitors. ES-Cu-induced cell death was rescued by TTM, whereas ferroptosis and apoptosis inhibitors failed to rescue ES-Cu-induced cell death. Cell viability was assessed by CCK-8 assay. **C-D.** FDX1-overexpressing AML cells were treated with ES-Cu in the presence or absence of TTM. Cell viability was assessed by CCK-8 assay. Data are presented as mean ± SD from three independent experiments. Two-way ANOVA was used in (C-D). $^{**}p < 0.01$, and $^{***}p < 0.001$ vs. control.

Table S1. Primers and shRNA sequences used in this study.

Gene	Primer Sequence
**The primer sequences for qRT-PCR.**	
FDX1	F: 5' TTCAACCTGTCACCTCATCTTTG -3'
	R: 5'- TGCCAGATCGAGCATGTCATT -3'
LIPT1	F: 5'-CAGGAACAGCTTCTAAGATCGGC-3'
	R: 5'- AGCAGTGGCATTGCTCCTGATC-3'
LIAS	F: 5'-TGGTGTGACTACTTCAGAACCT-3'
	R: 5'-GGAATAGGGCATGTGGATTTAGCA-3'
DLD	F: 5'-CCGAACTGATGTAAGTAAACGGTC-3'
	R: 5'-GCCCACGTATTTGAGTTCCGTA-3'
PDHA1	F: 5'-TGGTAGCATCCCGTAATTTTGC-3'
	R: 5'-ATTCGGCGTACAGTCTGCATC-3'
PDHB	F: 5'-AAGAGGCGCTTTCACTGGAC-3'
	R: 5'-ACTAACCTTGTATGCCCCATCA-3'
DLAT	R: 5'-CTGGCTCACAAAGCAGAGGA-3'
	R: 5'-GCCAACCCAAGCAACTTCAG-3'
ME1	F: 5'- GAGTGCTGACATCTGACATTGA-3'
	R: 5'- TTGGCTTCCGAAACACCAAAC -3'
Actin	F: 5'- CATGTACGTTGCTATCCAGGC -3'
	R: 5'- CTCCTTAATGTCACGCACGAT -3'
Luciferase	F: 5'-CTGGGACGAAGACGAACAC -3'
	R: 5'-GAAGACCTGCGACACCTG -3'
**The primer sequences for Chip-PCR.**	
ME1-RE	F: 5'- GCCTTAGTATGTGGATTC-3'
	R: 5'- GGAAAGCGTAGGGAAGGA-3'
p53	F: 5'- CCAGGGCAGCTACGGTTTC-3'
	R: 5'- CTCCGTCATGTGCTGTGACTG-3'
**Sequences of shRNA**	
FDX1-sh1 target seq	GCAATCACTGATGAGGAGAAT
FDX1-sh1	F: 5'- GCAATCACTGATGAGGAGAAT-3'
	R:5'-ATTCTCCTCATCAGTGATTGC-3'

FDX1-sh2 target seq	GATGCCAGACAATCCATTGAT
FDX1-sh2	F: 5'- GATGCCAGACAATCCATTGAT-3'
	R:5'-ATCAATGGATTGTCTGGCATC -3'
shME1 target seq	GCCCATGAACATGAAGAAATG
shME1	F: 5'- GCCCATGAACATGAAGAAATG-3'
	R:5'-CATTTCTTCATGTTCATGGGC -3'
sh-p53	F: 5'-GACTCCAGTGGTAATCTAC-3'
	R: 5'-GTACTCTCCTCCCCTCAAT-3'

Table S2. Clinical characteristics of AML patients with low and high serum Cu levels in our clinical cohort.

Patient characteristics	Low serum Cu level	High serum Cu level	X^2	p-value
n	58	68		
Age			0.09	0.76
<=60	43 (74.1%)	52 (76.5%)		
>60	15 (25.9%)	16 (23.5%)		
Sex			0.02	0.89
Male	34 (58.6%)	39 (57.4%)		
Female	24 (41.4%)	29 (42.6%)		
FAB classifications, n (%)			8.12	0.32
M0	1 (1.7%)	2 (2.9%)		
M1	7 (12.1%)	3 (4.4%)		
M2	19 (32.8%)	16 (23.5%)		
M3	3 (5.2%)	6 (8.8%)		
M4	6 (10.3%)	6 (8.8%)		
M5	21 (36.2%)	33 (48.5%)		
M6	1 (1.7%)	0 (0.0%)		
M7	0 (0.0%)	2 (2.9%)		

Using a chi-square test. The p-value was set at 0.05.

Table S3. Clinical characteristics of AML patients between FDX1-low and FDX1-high group in our clinical cohort.

Patient characteristics<br>n	Low expression<br>41	High expression<br>14	X^2	p-value
Age			2.90	0.09
<=60	16 (39.0%)	2 (14.3%)		
>60	25 (61.0%)	12 (85.7%)		
Sex			0.05	0.82
Male	31 (75.6%)	11 (78.6%)		
Female	10 (24.4%)	3 (21.4%)		
FAB classifications, n (%)			13.89	0.06
M0	0 (0.0%)	1 (7.1%)		
M1	1 (2.4%)	2 (14.3%)		
M2	15 (36.6%)	4 (28.6%)		
M3	1 (2.4%)	2 (14.3%)		
M4	3 (7.3%)	3 (21.4%)		
M5	19 (46.3%)	2 (14.3%)		
M6	1 (2.4%)	0 (0.0%)		
M7	1 (2.4%)	0 (0.0%)		

Using a chi-square test. The p-value was set at 0.05.
